# Mental Effort and Information-Processing Costs Are Inversely Related to Global Brain Free Energy During Visual Categorization

**DOI:** 10.3389/fnins.2019.01292

**Published:** 2019-12-05

**Authors:** Logan T. Trujillo

**Affiliations:** Department of Psychology, Texas State University, San Marcos, TX, United States

**Keywords:** brain free energy, mental effort, information-processing costs, visual categorization, electroencephalography

## Abstract

*Mental effort* is a neurocognitive process that reflects the controlled expenditure of psychological information-processing resources during perception, cognition, and action. There is a practical need to operationalize and measure mental effort in order to minimize detrimental effects of mental fatigue on real-world human performance. Previous research has identified several neurocognitive indices of mental effort, but these indices are indirect measures that are also sensitive to experimental demands or general factors such as sympathetic arousal. The present study investigated a potential direct neurocognitive index of mental effort based in theories where bounded rational decision makers (realized as embodied brains) are modeled as generalized thermodynamic systems. This index is called *free energy*, an information-theoretic system property of the brain that reflects the difference between the brain’s current and predicted states. Theory predicts that task-related differences in a decision makers’ free energy are inversely related to information-processing costs related to task decisions. The present study tested this prediction by quantifying global brain free energy from electroencephalographic (EEG) measures of human brain function. EEG signals were recorded while participants engaged in two visual categorization tasks in which categorization decisions resulted from the allocation of different levels of mental information processing resources. A novel method was developed to quantify brain free energy from machine learning classification of EEG trials. Participant information-processing resource costs were estimated via computational analysis of behavior, whereas the subjective expression of mental effort was estimated via participant ratings of mental workload. Following theoretical predictions, task-related differences in brain free energy negatively correlated with increased allocation of information-processing resource costs. These brain free energy differences were smaller for the visual categorization task that required a greater versus lesser allocation of information-processing resources. Ratings of mental workload were positively correlated with information-processing resource costs, and negatively correlated with global brain free energy differences, only for the categorization task requiring the larger amount of information-processing resource costs. These findings support theoretical thermodynamic approaches to decision making and provide the first empirical evidence of a relationship between mental effort, brain free energy, and neurocognitive information-processing.

## Introduction

Consider the extensive practice of a manual skill, undertaking a difficult exam, driving along a busy highway, or searching through a cluttered visual display. These activities engage perceptual, cognitive, and/or motor processes under varying levels of cognitive control to produce flexible, adaptive behavior (Schneider and Shiffrin, 1977; Shiffrin and Schneider, 1977). Engaging, maintaining, and controlling these processes requires different levels of *mental effort*, which may be operationally defined as a mediator between “the characteristics of a target task and the subject’s available information-processing capacity and …the fidelity of the information-processing operations actually performed, as reflected in task performance” (Shenhav et al., 2017, pp. 100–101). According to this view, task characteristics necessitate the executive allocation of limited neurocognitive information-processing resources for the successful completion of a task. Mental effort reflects those neurocognitive processes that control how much of an individual’s information-processing resources are actually allocated during task performance. As more mental effort is expended by an individual during a task, more information-processing resources are allocated up to the person’s maximum information-processing capacity. Mental effort is usually experienced as unpleasant, such that individuals are often reluctant to expend effort unnecessarily (Krebs et al., 2010; Padmala and Pessoa, 2011; Umemoto and Holroyd, 2014; Botvinick and Braver, 2015; Shenhav et al., 2017), although under certain conditions mental effort may be experienced as rewarding (Cacioppo and Petty, 1982; Eisenberger, 1992).

**FIGURE 1:**
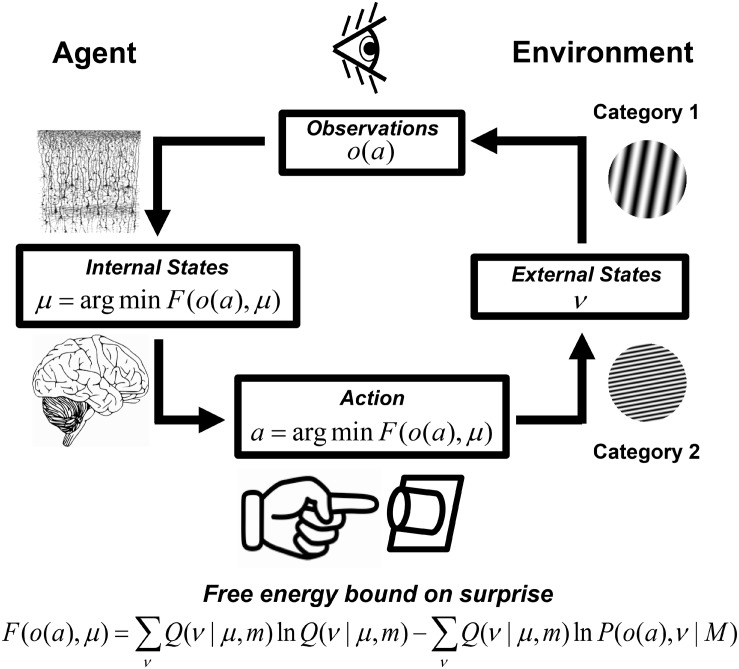
Approximate Bayesian-inference and free energy minimization in visual categorization. External world states ν encompass hidden causes of observations *o.* Observations may also be influenced by actions *a*, which change external world states and resultant observations. Brain free energy and surprise is minimized when (1) the brain, parameterized by internal neural states μ, approximately predicts the causes of observations according to a model *m* that partially-encodes an optimal model of the world *M*, or (2) when the brain’s actions change external world states and resultant observations to be in accordance with predictions based on a sub-optimal world model *m* encoded by the brain. For a detailed explanation, see Supplementary Material: The Free Energy Principle (FEP) for the Brain. Neural tissue slice figure element from Comparative Study of the Sensory Areas of the Human Cortex (p. 363) by S. Ramón y Cajal, 1899, Worcester, MA, United States: Clark University (Ramón y Cajal, 1899). Image is in public domain. Brain image (no title, author unknown), uploaded July 8, 2014, retrieved January 15, 2019 from https://pixabay.com/vectors/brain-intelligence-science-mind-312007/. Image is in the public domain via a CC0 license.

There is a practical need to operationalize and measure mental effort. Excessive mental effort typically induces mental fatigue that negatively affects real-world human performance (Grandjean, 1979; Parasuraman et al., 2008; Kato et al., 2009; Galy et al., 2012; Zhao et al., 2012; Witkowski et al., 2015). Thus accurate measurement of mental effort will inform efforts to minimize mental fatigue in human operators. Several neurocognitive indices have been put forward to index mental effort (e.g., response times, avoidant preferences, pupil diameter, facial electromyography, and frontocortical activity, etc.), however, these measures are often sensitive to experimental demands or general factors such as sympathetic arousal (Shenhav et al., 2017). The present study investigated a system property of the brain called *free energy* that in theory is directly sensitive to the information-processing resource costs allocated through mental effort. The concept of free energy originated in thermodynamic physics where it is a measure of the work (or useful energy) a physical system can exert after accounting for internal energy losses due to heat (Huang, 1987). Brain free energy is an information-theoretic generalization of this concept that reflects the brain’s *surprise* – the difference between the brain’s current and predicted states (Pio-Lopez et al., 2016); see Figure 1. In this context, free energy acts as a motivating influence for the brain in that the latter seeks to minimize its free energy (and thus its surprise) in order to maintain physiological homeostasis (see section “The Free Energy Principle (FEP) for the Brain”). The minimization of the brain’s free energy corresponds to a process of approximate Bayesian inference that has important consequences for perception, cognition, and action (Feldman and Friston, 2010; Friston, 2010; Friston et al., 2010, 2015, 2016; Pio-Lopez et al., 2016; Parr et al., 2018). The process of brain free energy minimization has been termed the FEP (Friston, 2010).

**FIGURE 2:**
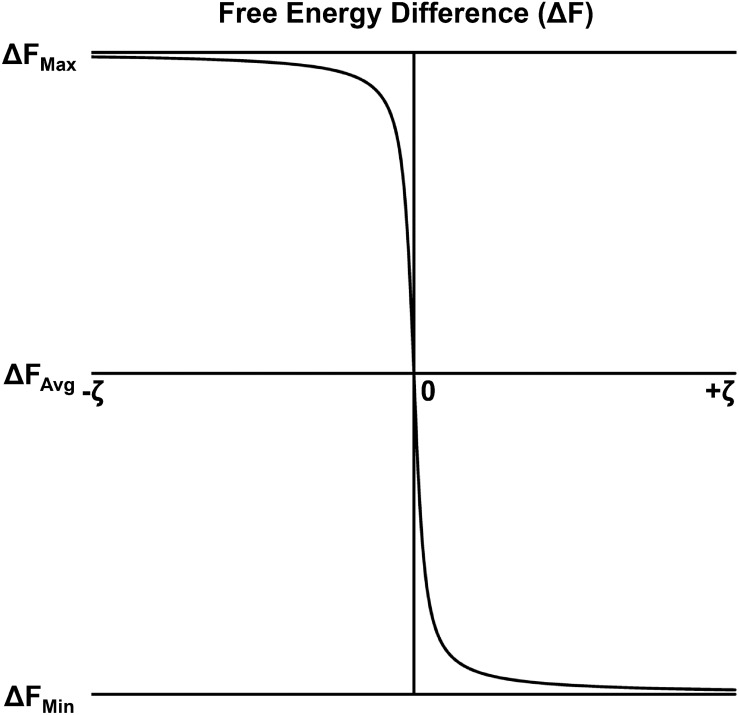
Free energy difference as a function of resource/confidence parameter ζ for the visual categorization task proposed here. The free energy difference displayed here was computed following a modification of the calculation of Ortega and Braun (2013), under the assumption that the relevant probabilities are described by Boltzmann distributions.

The theoretical sensitivity of brain free energy to mental resource costs is based in thermodynamical approaches to modeling bounded rationality (Ortega and Braun, 2013). Bounded rationality is the idea that real-world decision makers have limited information-processing resources and thus are unable to perform the total amount of deliberation necessary to make an optimum or perfectly rational decision (Simon, 1956, 1972, 1984; Aumann, 1997). Instead, real-world decisions are based on the limited set of deliberations attainable given the available level of information-processing resources. In this approach, decision-makers (realized as embodied brains) are modeled as thermodynamic systems described by probability distributions that change as mental information-processing takes place. However, this information-processing incurs a cost in terms of the computational resources necessary to reach a decision. The actual decision that is made reflects a trade-off between any gains in utility or value resulting from the decision and the costs of information-processing underlying the decision. (In analogy to the thermodynamic physics definition of free energy, the utility of a decision plays the role of internal energy and information-processing costs play the role of heat.) It can be shown that this trade-off can be mathematically described in terms of a (negative) free-energy difference of a decision maker across an information-processing cycle (Ortega and Braun, 2013), with the allocated level of information-processing resources described in terms of an “inverse temperature” parameter ζ for the relevant probability distributions. Importantly, there is a reciprocal relationship between the ζ parameter and free energy differences (Ortega and Braun, 2013; Friston et al., 2016); see Figure 2. Therefore, in so far as mental effort reflects the executively controlled allocation of information-processing resources, then it should also have a similar relationship to differences in brain free energy.

The goal of the present study was to empirically test this predicted relationship in a task context requiring visual category decisions; the objective was not to devise a single study that could decide between other theories and the thermodynamic approach to bounded rational decision making, but instead to provide evidence to either support or falsify the predictions of this theory as well as the FEP. Visual categorization is a fundamental cognitive process in which visual objects are mentally placed into classes or groups on the basis of similar perceptual characteristics of different object properties (Goldstone and Kersten, 2003; Rips et al., 2012). Categorization was chosen as the task context for two reasons. First, categorization allows for the experimental manipulation of task characteristics that incur different levels of information-processing costs. Object categories can be easily defined to overlap with each other in terms of diagnostic object features in order to produce different levels of neurocognitive representational interference; the information-processing limits that emerge from this interference are called *representational capacity constraints* (Shenhav et al., 2017). Cognitive control is then necessary to reduce this interference in order to yield satisfactory task performance, with higher degrees of overlap/interference requiring a greater degree of controlled information-processing to resolve (Shenhav et al., 2017); see section “Experimental Methods, Categorization Task”. Second, the ability to categorize objects is crucial for organisms to survive in their environment (Ashby and Maddox, 1997), where they must make life-sustaining decisions on the basis of their perceptions. Understanding the impact of mental effort on categorization-related information-processing could inform efforts to improve human decision making and cognitive control (Shenhav et al., 2017).

In the present study, a novel procedure was developed to estimate brain free energy differences from machine learning classification of participant electroencephalographic (EEG) recordings of global brain states during the perception of simple visual categories defined by an implicit integration of stimulus orientation and spatial frequency (2-AFC categorization of Gabor stimuli; see sections “Analytical Methods, Global Brain Free Energy Difference Quantification” and “Experimental Methods, Categorization Task”). The global brain free energy differences estimated in this manner were then related to estimates of participant information-processing resource allocation; the latter were taken as an objective proxy for the mental effort expended during the visual categorization task. Information-processing resource costs were indexed via the inverse temperature parameter ζ. The parameter was estimated from each participant’s visual categorization behavior by application of a softmax perceptual decision-making model (Reverdy and Leonard, 2016) with a mathematical form that minimizes the free energy difference of a bounded-rational decision maker (Ortega and Braun, 2013); see section “Analytical Methods, Resource Allocation Parameter Estimation”. Participants performed two different categorization tasks that theoretically implemented different levels of representational capacity constraints and thus required the expenditure of different information-processing resource costs for successful task performance (see section “Experimental Methods, Categorization Task”). The following predictions were then tested based on the theoretical reciprocal relationship between inverse temperature parameter ζ and free energy: positive brain free energy differences would negatively correlate with parameter ζ (Figure 2); and positive brain free energy differences would be smaller, and parameter ζ would be larger, for the visual categorization task that required the expenditure of a larger versus smaller amount of information-processing resource costs. These predictions were tested by correlating global brain free energy differences with the estimated ζ parameters across individual participants and by comparing free energy across the two visual categorization tasks.

Positive global brain free energy differences and the ζ parameter were also related to participant ratings of subjective mental workload in order to index the subjective expression of mental effort (Shenhav et al., 2017); see section “Experimental Methods, Subjective Assessment of Mental Workload”. This relationship was predicated on the finding that people subjectively experience mental effort as psychological “work” in proportion to the actual effort with which they engage in a task (Kantowitz, 1987). To the extent that mental effort reflects the subjective expression of information-processing resource allocation, ratings of mental workload should positively correlate with the ζ parameter values and negatively correlate with positive differences in global brain free energy.

## Materials and Methods

In this section, the basic conceptual framework and mathematical formalism of the FEP is described first, including its formal relationship to information-processing costs and perceptual categorization. This is followed by a description of the experimental and analytical methods used to apply the FEP to the study of mental effort during visual categorization.

### The Free Energy Principle (FEP) for the Brain

#### Free Energy Minimization and Approximate Bayesian Inference

The FEP is a general theoretical principle that has been proposed to provide a unified account of brain functioning (Friston, 2010). This principle originates in the observation that adaptive agents such as embodied brains seek to minimize surprise – the difference between a brain’s current and predicted states – in order to maintain a systemic homeostasis in the face of destabilizing influences in the environment (Friston, 2010; Pio-Lopez et al., 2016). One way the brain achieves this is by organizing itself in a manner that reflects the causal and structural regularities of its environment so as to predict and oppose environmental changes that disrupt homeostasis (Friston, 2010, 2012). That is, the brain’s organization represents a *generative model m* of its environment that it uses to generate data or observations *o* from hidden environmental variables ν that generate or cause the observations but are not directly evident from the pattern of observations. These hidden states are inferred by the brain and are represented via internal neural states in a manner that minimizes an upper bound on surprise called *free energy* – a higher-order probabilistic function of the brain’s observed states and its internal representation of the causes of observations, given the brain’s generative model; see Figure 1. Free energy may be expressed as a higher-order function of observations and causes as (Friston, 2010; Friston et al., 2014):

(1) $$F\,\left(o,\mu \right)=\sum_{\nu }Q\,\left(\nu |\mu ,m\right)\,\ln Q\,\left(\nu |\mu ,m\right)-\sum_{\nu }Q\,\left(\nu |\mu ,m\right)\,\ln P\,\left(o,\nu |M\right)$$

where *P(o*,ν*| M)* is the *generative model distribution* describing the joint probability of observations and their causes given the brain’s theoretically best possible (i.e., “correct” or “true”) encoding of this information, an optimum generative model denoted by *M*. The distribution *Q(*ν*|*μ*,m)* is called the *recognition distribution* and reflects a probabilistic neural representation of the causes of observations conditional on a distribution parameter represented within the brain by internal neural states μ.

The free energy bound on surprise arises by treating the brain as a Bayesian agent that transforms prior beliefs into posterior beliefs according to a posterior distribution *P(*ν*|o,m)* described by Bayes’ rule, an approach called *the Bayesian brain hypothesis* (Lee and Mumford, 2003; Knill and Pouget, 2004; Doya et al., 2007). In many situations, a direct computation of the true posterior *P(*ν*|o,M)* is computationally intractable because the causes of observations are hidden variables and the number of possible causes of observations can be very large (Dayan et al., 1995; Pio-Lopez et al., 2016). The FEP approach circumvents this by assuming that the brain embodied as an agent minimizes its free energy by performing approximate Bayesian inference, which may be carried out in two ways. First, the brain may optimize its representations about the causes of its observations by optimizing the recognition distribution *Q(*ν*|*μ*,m)* to be as close as possible to *P(*ν*|o,M)*; see Figure 1. Given that this internal representation is in part constrained by the brain’s organization, such an optimization may also involve the brain changing its organization in order to encode a better approximation of the optimum generative model *m*. Second, an embodied brain agent may minimize its free energy by acting on the world in order to change observations in accordance with its (sub-optimal) predictions, where such actions “[enforce] a sampling of [observed] data that is consistent with the current representation … [in order to] minimize prediction error” (Friston, 2010, p. 128); see Figure 1. In this case, actions influence observations, *o* = *o(a)*, and free energy may be expressed as (following Friston et al., 2015; Pio-Lopez et al., 2016; Gershman, 2019),

(2) $$F\,\left(o\,\left(a\right),\mu \right)=\sum_{\nu }Q\,\left(\nu |\mu ,m\right)\,\ln Q\,\left(\nu |\mu ,m\right)-\sum_{\nu }Q\,\left(\nu |\mu ,m\right)\,\ln P\,\left(o\,\left(a\right),\nu |M\right)$$

Minimization of free energy with respect to actions is called *active inference* (Friston et al., 2015; Pio-Lopez et al., 2016; Gershman, 2019).

#### Free Energy and Perceptual Categorization

In the present study, the free energy *F(o*,ν*)* of global states of the human brain were quantified during the perception of simple visual categories. In the original formulation of the FEP, *sensations* are the observations about which the brain seeks to minimize its free energy estimate of surprise, and the relevant hidden variables reflect different physical features of an object (e.g., orientation of line segments, spatial frequency, etc.). However, causes can also be categorical in nature (Friston, 2005). In the present study, the observations under consideration were *category perceptions*, in which perceptual objects are perceived to be members of discrete categories and/or referents of *concepts* – abstract mental representations of the general properties and structure of object classes that may also serve to structure and influence perceptions (Goldstone and Kersten, 2003; Rips et al., 2012). In some Bayesian approaches to categorization (e.g., Shi et al., 2010), hidden variables reflect the concepts that refer to different categories (where concepts are operationalized as the assignment of semantic labels to the categories); in this case the posterior distribution *P(*ν*|o,m)* indexes the probability that a category label describes an object, given the object’s perceptual characteristics. Thus the theoretical FEP framework can also be used to describe how the brain approximates this posterior distribution of category labels via free energy minimization of surprise. In this case, the surprise to be minimized reflects the difference between the predicted and correct or “true” category label of an object. These are quantities for which probability distributions can be estimated from the *a priori* knowledge of the stimulus category on each trial and probabilistic estimates of the brain’s representations of its category perceptions to yield an empirical measure of free energy (see section “Analytical Methods, Global Brain Free Energy Difference Quantification”).

### Free Energy Differences and Information-Processing Costs

In the thermodynamic approach to bounded rationality, decisions reflect a trade-off between gains in utility and the costs of information-processing. In the specific case where the relevant statistical distributions are Boltzmann distributions, it can be shown (Ortega and Braun, 2013) that this trade-off takes the mathematical form of a *negative free-energy difference*,

(3) $$-\Delta \,F\,\left(q\,\left(\nu \right)\right)=\mathrm{Expected}\,\mathrm{Utility}-\mathrm{Information}\,\mathrm{Processing}\,\mathrm{Cost}=\sum_{\nu }q\,\left(\nu \right)\,U\,\left(\nu \right)-\frac{1}{\zeta }\,\sum_{\nu }q\,\left(\nu \right)\,\ln\frac{q\,\left(\nu \right)}{p_{0}\,\left(\nu \right)}$$

Here ν represents an individual decision outcome out of a set of possible decision outcomes, *U(*ν*)* quantifies the utility for each possible outcome, *p_0_(*ν*)* is a prior distribution that reflects the initial information state of the decision maker, *q(*ν*)* is the final information state, and ζ is a parameter that reflects the allocated level of information-processing resources.

The mathematical form of Eq. 3 is analogous to the thermodynamic physics definition of free energy (see “Introduction” section). The first term in Eq. 3 reflects the expected utility gain (or loss) from the decision and is mathematically represented as an expected energy. The second term reflects a decision maker’s computational costs of changing from an initial to final information state and is mathematically represented as the relative entropy of the two states (in analogy to thermodynamic entropy which reflects energy loss via heat). Intuitively, Eq. 3 reflects the net amount of mental “work” performed by the decision maker after subtracting the costs to implement the decision from the total amount of mental “work” exerted. According to the FEP, the brain seeks to minimize its free energy (and thus surprise) about the outcomes of its decisions. As Eq. 3 represents a negative free energy difference, free energy minimization (a decrease in positive free energy from a maximum to a minimum value) is equivalent to the maximization of this negative difference (i.e., an increase in negative free energy from a minimum to a maximum value). This extremization occurs when the distribution of the final information state *q(*ν*)* takes the approximate form of a final prior distribution *p(*ν*)* that represents an equilibrium state (i.e., the actual decision).

The particular mathematical form of the (negative) free energy difference expressed by Eq. 3 reflects the case for Boltzmann-type of statistical distributions and utility functions that reflect the internal energy of the decision maker (Ortega and Braun, 2013). However, decisions resulting from approximate Bayesian inference typically involve the use of more general statistical distributions and utility functions that reflect the brain’s optimum generative model of its environment. From the perspective of the thermodynamic approach to bounded rationality, ν can also be interpreted as representing a decision outcome about the hidden variables. For example, assume general distributions for a decision maker’s initial and final information states *P_0_(*ν*| m)* and *Q(*ν*|*μ*,m)* entailed by their generative model *m*. Assume the decision’s utility function to take the form *U(o*,ν*| M)* = *ln(P(o|*ν*,M))* = *ln(P(o*,ν*| M)/P(*ν*| M))*, as entailed by the optimum generative model *M*. Then in the case when the true prior distribution is known by the decision maker and is constant (the case considered in the categorization task utilized here; see Supplementary Material: Free Energy Differences Under Constant Prior), the positive free energy difference is given as

(4) $$\Delta \,F\,\left(o,\mu \right)=\mathrm{Information}\,\mathrm{Processing}\,\mathrm{Cost}-\mathrm{Expected}\,\mathrm{Utility}=\sum_{\nu }Q\,\left(\nu |\mu ,m\right)\,\ln Q\,\left(\nu |\mu ,m\right)-\sum_{\nu }Q\,\left(\nu |\mu ,m\right)\,\ln P\,\left(o,\nu |M\right)$$

It should be clear that Eq. 4 is equivalent to Eq. 1; this equivalence illustrates that, in the case of known constant priors, absolute free energy levels may also be considered to be free energy differences relative to a zero baseline; see Supplementary Material: Free Energy Differences Under Constant Prior.) The free energy difference expressed by Eq. 4 is always greater than or equal to the brain’s surprise and thus is always non-negative in value (see Supplementary Material: Free Energy Differences Under Constant Prior). Moreover, in this general case, the resource parameter ζ is implicit within the probability distributions defining Eq. 4, where it behaves as an “inverse temperature” that parameterizes the precision of an individual’s posterior beliefs (Friston et al., 2014, 2016). The effect of this implicit parameter is to restrict *Q(*ν*|*μ*,m)* to a subset of possible distributions, which limits rational information-processing (Ortega and Braun, 2013).

Theoretically, there is an inverse relationship between the resource parameter ζ and differences in free energy (Ortega and Braun, 2013; Friston et al., 2016); see Figure 2. As allocated information-processing resources ζ increase, the magnitude of the free energy difference decreases (i.e., positive free energy decreases to a minimum and negative free energy increases to a maximum). In contrast, as allocated information-processing resources ζ decrease, the magnitude of the free energy difference increases (positive free energy increases toward a maximum and negative free energy decreases to a minimum). This inverse relationship between ζ and Δ*F(o*,μ*)* is explicitly expressed in Eq. 3 for the case of Boltzmann-type of distributions. In the general statistical case expressed by Eq. 4 where ζ is implicit within the probability distributions, the value of this parameter must be inferred from the data via computational modeling. Here, ζ was computationally estimated from participant categorization behavior using a softmax perceptual decision-making model (Reverdy and Leonard, 2016) with a mathematical form that minimizes the free energy difference of a bounded-rational decision maker (Ortega and Braun, 2013); see section “Analytical Methods, Resource Allocation Parameter Estimation”.

### Experimental Methods

#### Participants

Fifty eight Texas State University students participated for course credit or monetary payment. However, the data of 10 participants was not included in the final analysis due to technical recording errors (*n* = 2), excessive sleepiness (*n* = 1), excessive data loss due to ocular artifacts (*n* = 6), and excessive non-responses during task performance (*n* = 1). Hence the final sample consisted of forty eight participants (29 female, 19 male, mean age = 19.5 years, age range = 18–26). This study was carried out in accordance with the recommendations of the Institutional Review Board at Texas State University with written informed consent from all participants. All participants gave written informed consent in accordance with the Declaration of Helsinki. The protocol was approved by the Institutional Review Board at Texas State University.

#### Categorization Task

**FIGURE 3:**
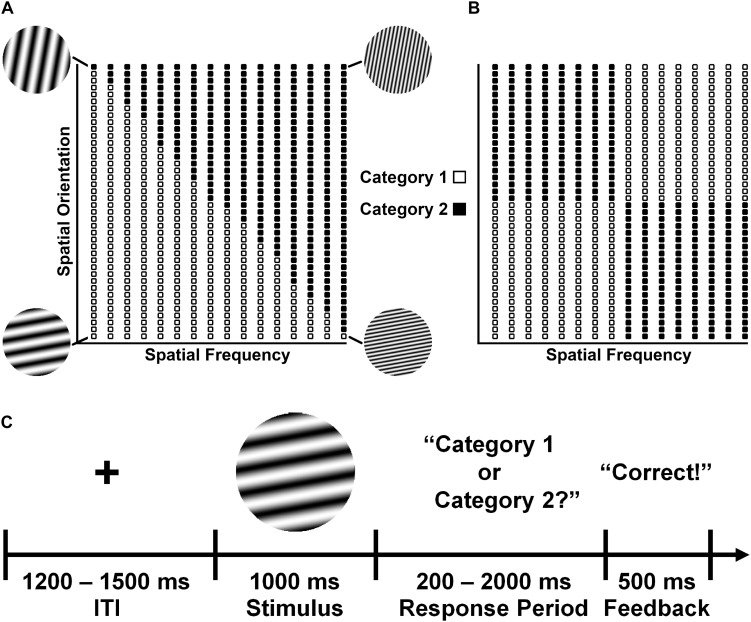
Example category distributions for the **(A)** II categorization task and the **(B)** RB categorization task. Examples shown illustrate one particular assignment of categories to regions of the stimulus space given to half of the participants; the remaining participants received the opposite assignment. **(C)** Basic categorization task protocol. A fixation cross was presented for a variable interstimulus interval (ITI) at the center of a computer screen, followed by the stimulus for 1000 ms. The stimulus was then removed and the participant was visually queried about the stimulus category. The subject had a maximum of 2000 ms to respond “Category 1” or “Category 2” by pressing one of two buttons on a computer mouse. This was followed by visual feedback (“Correct”, “Incorrect”, or “No Response”) for 500 ms before a new trial begun. Additional task details may be found in the main text and the Supplementary Material.

Participants performed two visual categorization tasks that differed in terms of difficulty and the complexity of the stimulus category space (Figure 3); these tasks were modifications of previous task paradigms used to study visual category-learning (Morrison et al., 2015). Participants categorized circular sine-wave gratings (Gabor patches) into two categories defined by the spatial frequency and orientation of the gratings.

The dependency of category membership on these visual features differed between the two tasks. In the *information integration (II) task* (Figure 3A), the stimuli were divided into two categories defined by a diagonal decision boundary that required participants to integrate frequency and orientation information in a manner that was not amenable to a simple rule that could be verbalized. The sign (±) of the decision boundary slope was balanced across participants. For the *rule-based (RB) task* (Figure 3B), the stimuli were divided into two categories based on vertical and horizontal decision boundaries that required participants to psychologically integrate frequency and orientation information according to a simple multidimensional rule that could be easily verbalized (e.g., “category A stimuli are oriented more vertically and have lower frequencies or are oriented more horizontally and have higher frequencies; category B stimuli are characterized by the opposite pattern”).

Crucially, the visual categories in each task overlapped with each other in terms of spatial frequency and orientation. Such stimulus feature overlap is well-known to produce representational capacity constraints via interference among task-related neurocognitive representations that requires additional information-processing resources to resolve (Shenhav et al., 2017). However, it was hypothesized here that the level of resources necessary for visual categorization would be greater for the RB task than for the II task (see “Introduction” section). There were two bases for this hypothesis. First, the structure of visual feature overlap was more complex for the RB Task than the II task. Second, the category structures of these tasks are known to engage distinct neurocognitive systems that have different representational characteristics and information-processing requirements (Nomura et al., 2007). Categorization based on verbalizable rules (the RB Task) is known to be mediated by an explicit representational system that requires effortful attention for its operation, whereas categorization based on non-verbalizable criteria (the II Task) is mediated by an implicit system that operates automatically (Maddox and Ashby, 2004).

A schematic of a typical task trial is shown in Figure 3C; trial description is given in the Figure 3C caption. Prior to task performance, participants were familiarized with task procedures and received explicit instruction about the category structure of each task. Participants were shown the prototypes of each category and, for the II task, they were also shown additional stimulus examples. Participants were also told that they would be presented with equal numbers of stimuli from each category. Task order was balanced across participants. For additional task information, see Supplementary Material: Experimental Methods – Technical Details.

#### Subjective Assessment of Mental Workload

The subjective experience of mental effort was quantified via the *Workload Profile (WP)* (Tsang and Velazquez, 1996), a psychometric instrument that indexes the subjective expression of mental effort along eight dimensions (perceptual/central processing, response processing, spatial processing, verbal processing, visual input modality, auditory input modality, manual output modality, speech output modality). The WP has been shown to be a highly valid, sensitive, and diagnostic index of mental workload that is well suited to assess the different cognitive demands, attentional resources, and difficulty levels of cognitive and motor tasks (Valdehita et al., 2004). Each participant’s WP dimension scores were added to yield a global workload score; for the specific WP version used here, see Supplementary Material: Experimental Methods – Technical Details.

#### EEG Recording and Pre-processing

**FIGURE 4:**
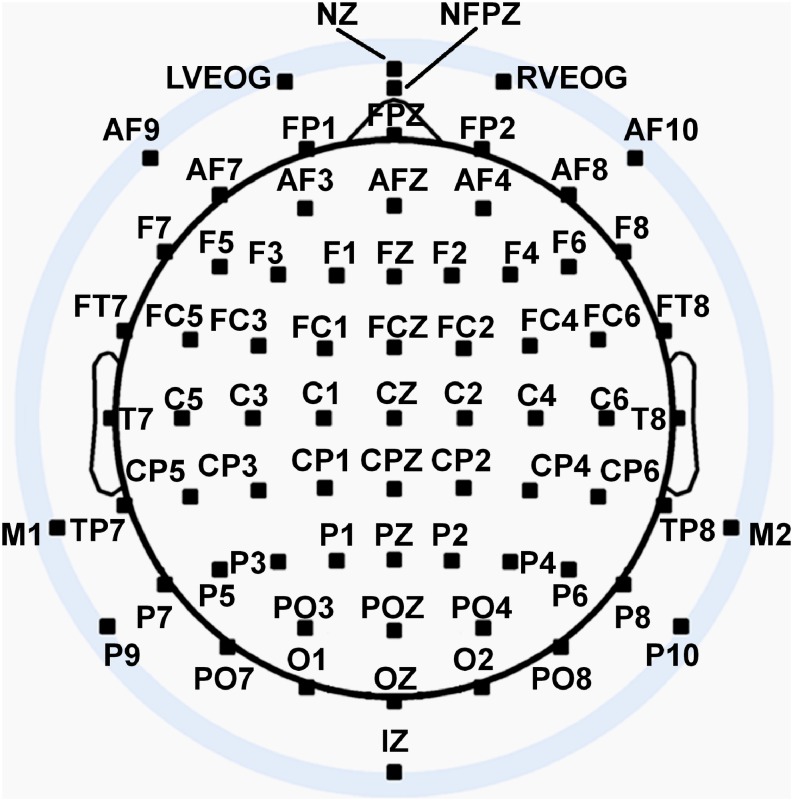
Extended 10–20 scalp locations of EEG recording electrodes. Sites outside the radius of the head represent locations that are below the equator (FPZ-T7-T8-OZ plane) of the (assumed spherical) head model. Figure adapted from Trujillo et al. (2017) with permission of the authors.

Seventy two channels of continuous EEG signals were recorded using a Biosemi Active II amplifier system (24-bit DC mode, input sampling rate of 2048 Hz downsampled online to 256 Hz) and active Ag/AgCl electrodes either mounted in an electrode cap or via freestanding electrodes. Recording sites included international 10/5 system locations (Jurcak et al., 2007) and the inferior orbits of the eyes (Figure 4). EEG signals were recorded with respect to a common mode sense (CMS) electrode located between sites PO3 and POZ. Half-cell potentials of the electrode/gel/skin interface were kept between ±40 mV, following standard recommendations for the Active II system. EEG data were imported offline into the MATLAB 2017b computing software environment (The Math Works, Inc., Natick, MA, United States) using the EEGLAB toolbox (Delorme and Makeig, 2004) for MATLAB, with all subsequent analysis performed via in-house scripts utilizing EEGLAB functions. Standard EEG preprocessing procedures (Picton et al., 2000) were used including artifact-scoring, bad channel interpolation, average reference transformation, trial epoching from −200 ms–1000 ms relative to stimulus onset, bandpass filtering (0.1–30 Hz), and baseline-correction to the 200 ms pre-stimulus interval. For additional technical detail about EEG pre-processing, see Supplementary Material: Experimental Methods – Technical Details.

#### General Procedure

After consent, participants underwent setup for EEG recording, during which participants completed several questionnaires indexing demographic and health information, sleep quality/quantity, emotion/mood states, and current attentional states. The results of these questionnaires are irrelevant to the hypotheses tested in this paper and are not reported here. After completion of the EEG setup, participants underwent an 8 min period of resting state EEG recording. As resting state brain dynamics are not the focus of this paper, this data is not reported here; a spectral and information-theoretic analysis of a portion of the resting state EEG data has been reported previously (Trujillo et al., 2017). Following recording of the resting state EEG, EEG data was then recorded while participants performed the two visual categorization tasks that were the focus of the present study. Participants immediately completed the WP questionnaire to subjectively estimate their workload after each task.

### Analytical Methods

#### Statistical Analysis of Categorization Task Performance and Mental Work

Statistical assessment of categorization task performance and mental workload (indexed via global WP score) was performed using non-parametric permutation-based ANOVAs (LeFleur and Greevy, 2009; 5000 permutations) implemented via EEGLAB. Categorization accuracy versus chance was analyzed separately for each task via one-way repeated measures analysis of variance (ANOVA). A one-way repeated measures ANOVA was also used to assess potential accuracy differences between categorization tasks. Response times for participants to indicate categorization decisions were analyzed via two-way repeated measures ANOVA with factors of Categorization Task (RB, II) and Categorization Accuracy (Correct, Incorrect). Between-task differences in global WP scores were assessed via one-way repeated measures ANOVA. All *post hoc* multiple comparisons were corrected to control the False Discovery Rate to be less than or equal to 0.05 (Benjamini and Yekutieli, 2001); corrected *p*-values are indicated as such in the text.

#### Global Brain Free Energy Difference Quantification

**FIGURE 5:**
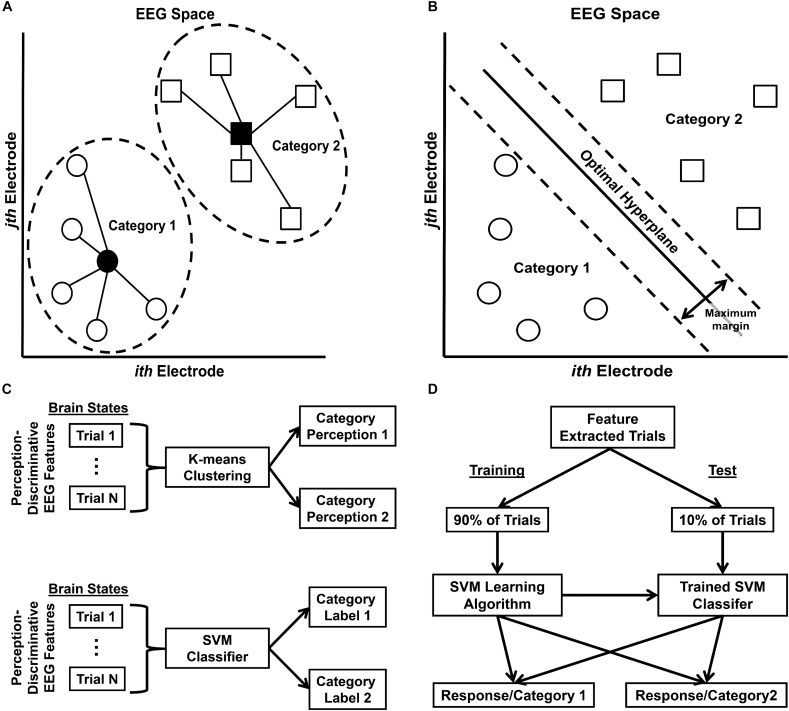
Free energy probability distribution computation. **(A)** Schematic representation of *K*-means clustering classifier in EEG data feature space (two dimensions shown for ease of visualization). Filled-in shapes indicate cluster prototypes; lines indicate distances from prototype. **(B)** Schematic representation of Support Vector Machine (SVM) classifier in EEG data feature space. **(C)** Schematic of general classification procedure for K-means clustering classification (top row) and SVM classification (bottom row). **(D)** Schematic of stratified 10-fold cross-validation SVM classifier training and test procedure. This procedure was performed for all possible 10-fold partitions such that all trials were eventually classified at test. Note that although the feature extraction was not incorporated into the SVM cross-validation procedure (i.e. tested trials were transformed via a CSP matrix computed from all trials, rather than only training trials), any effect on classifier generalization error was likely minimal; see Supplementary Material: Analytical Methods – Technical Details for further discussion.

The quantification of a brain free energy difference requires estimation of two probability distributions (see Eq. 1): the optimum generative model distribution *P(o*,ν*|M)* describing the true joint probability of optimal category perceptions *o* and their categories ν given an optimum generative model *M*, and the recognition distribution *Q(*ν*|*μ*,m)* describing the probability of true category labels ν given activation of a neural representation μ that parameterizes the distribution as entailed by the brain’s generative model *m*. These distributions were estimated for each participant (Figure 5) from their EEG-indexed brain responses by application of machine learning classification algorithms. The rationale here is that the classifiers provide an objective way to determine what brain state patterns encode information about a given class (e.g., category perceptions), under the assumption that trials classified into a given class contain a greater degree of information about that class than the opposite class (Haxby et al., 2014; Stewart et al., 2014). The brain free energy quantification procedure involved three steps:

#### Step 1: Estimating the Generative Model Probability Distribution

The generative model distribution *P(o*,ν*| m)* describes the brain’s model of its environment that it uses to generate observations *o* from hidden environmental variables ν that cause the observations. Typically the estimation of a generative model involves explicit assumptions about the distribution of the perceptual features necessary to create the observations (e.g., spatial frequencies, orientations) and how those features are perceptually partitioned into categories (Ashby and Maddox, 1993; Nomura and Reber, 2012). Here a simpler approach was taken that utilized a generative model derived under the assumption of a noise-free optimal categorizer and perceiver with perfect knowledge of how category perceptions map to category labels and the ability to perfectly discriminate among all the different possible perceptual features of the stimuli. Under this assumption, there is a one-to-one mapping between the optimal category perception of each stimulus and their category labels (e.g., see Figure 3) such that,

(5) $$P\,\left(o|\nu ,M\right)=P\,\left(\nu |o,M\right)=\delta_{o\,\nu }$$

and

(6) $$P\,\left(o,\nu |M\right)=P\,\left(o|\nu ,M\right)\,P\,\left(\nu \right)=P\,\left(\nu |o,M\right)\,P\,\left(o\right)=\delta_{o\,\nu }\times 0.5$$

where *P(o)* = 0.5 = *P(*ν*)* as the latter was set in the categorization task (see section “Experimental Methods, Categorization Task”). The implications of this choice of generative model will be discussed in the “Discussion” section.

#### Step 2: Estimating the Recognition Probability Distribution

The recognition probability distribution *Q(*ν*|*μ*,m)* reflects a conditional mapping between causes ν and neural representations μ that parameterize the distribution. The question raised here is what does μ represent and how can it be estimated? The answer to this question arises from the logical necessity that when free energy is minimized, *Q(*ν*|*μ*,m)* ≈ *P(*ν*|o,M)*. Hence if the brain of a perceiver minimizes its free energy during category perception, then the information encoded by neural state μ should approximately reflect the information encoded by the brain about its category perceptions because the true posterior encodes the probability of a true stimulus category conditional on the optimal category perceptions *o*. Thus to a first approximation, *Q(*ν*|*μ*,m)* was estimated by identifying the brain’s representation of its category perceptions and then using these representations to predict the true category labels of the stimuli. This allowed the computation of the approximate empirical posterior probability *Q(*ν*|*μ*,m)* that a classified EEG trial reflected true category brain state ν given category perception-specific brain state μ. A defense of this procedure will be given in the “Discussion” section.

In order to identify the brain’s representation of its observations, EEG trials underwent a feature extraction procedure, where the features were diagnostic physical information present in the EEG signals over the post-stimulus interval (0–1000 ms) of a trial. Here the common spatial patterns (CSP) method (Koles, 1991; Müller-Gerking et al., 1999; Ramoser et al., 2000) was used to extract sets of topographic spatial EEG patterns that maximally discriminated between the two possible category perceptions as indicated behaviorally by a participant. EEG trials were separated into one of two groups associated with a specific reported category perception; these groups were then entered into the EEG feature extraction procedure. It was assumed that the resulting spatial patterns reflected the neural representations specific to each category perception. (Feature extraction also provides an additional advantage of removing uninformative features and decreasing the chance of classifier overfitting by reducing the ratio of features to trials; Pereira et al., 2009). Following Ramoser et al. (2000), the CSP spatial patterns were used to create multidimensional feature vectors **f***_*CSP*_* for each EEG trial as follows:

(7) $$\begin{array}{c} f_{i}=\log\left(\frac{v\,a\,r\,\left(Z_{i}\right)}{\sum_{i=1}^{N_{r\,a\,n\,k}}v\,a\,r\,\left(Z_{i}\right)}\right)\\ f_{C\,S\,P}=\left[f_{1},f_{2},\ldots ,f_{N_{r\,a\,n\,k}}\right] \end{array}\}$$

where *Z*_*i*_ is the *i-th* activation time course of a given CSP pattern over an EEG trial and *N*_*rank*_ is the rank of the data matrix covariance matrix estimated by the CSP method. The CSP algorithm was applied after first decomposing the EEG data into independent subsets of variation via independent components analysis (ICA) (Stewart et al., 2014). ICA-transformation reduces the effects of EEG data interdependencies and noise on data covariance matrix estimation by the CSP method (Yger et al., 2015).

The CSP feature vectors were then used for K-means clustering and support vector machine (SVM) classification of EEG trials in order to compute the estimate of *Q(*ν*|*μ*,m)*. *K*-means clustering is an unsupervised machine learning algorithm that partitions data observations into *k* clusters (Figure 5A), where each observation belongs to the cluster with the nearest mean or cluster prototype (Forgy, 1965); see Figure 5C, top row, for a schematic of the K-means clustering procedure. This classifier was used to classify EEG trials exhibiting category perception-discriminative CSP brain patterns into one of two possible perceptual states. This created an index of the predicted category perception on each EEG trial that was then used to sort trials according to category perception after SVM classification (see below). SVMs are supervised classification algorithms that search for an optimal hyperplane separating data into two classes (Cortes and Vapnik, 1995; Christianini and Shawe-Taylor, 2000); boundaries between the classes are created by maximizing a margin around the optimal hyperplane (Figure 5B). This allowed the computation of the conditional posterior probability *Q(*ν*|*μ*,m)* that a classified EEG trial reflected category ν given category perception-specific brain state μ. These conditional probabilities were averaged across trials according to the category perception trial index created via K-means clustering to produce a final estimate of *Q(*ν*|*μ*,m)*; see Figure 5C, bottom row, for a schematic of the SVM classification procedure.

Ten-fold stratified cross-validation was used to train and test the SVM classifiers in order to reduce overfitting and ensure classifier generalizability (Figure 5D). As both the SVM cross-validation data partitioning and the initial *K*-means cluster centroids were determined randomly for each classifier, both classifications were performed 200 times for each participant. This yielded 200 separate estimates of *K*-means-based trial indices for the perceptual states, which were then used to sort and average the conditional probabilities computed on a corresponding SVM estimate. The final *Q(*ν*|*μ*,m)* estimate for each participant was then taken as the average over the 200 separate SVM-based estimates obtained from each participant’s data. The SVM application for each individual estimate also yielded an index of predicted category labels on each trial, which were used to determine spatiotemporal EEG patterns associated with the different free energy states (see section “Analytical Methods, Estimation of Free Energy Difference-Related Brain Responses”). This combined stratified cross-validation/bootstrapping procedure also mitigated any distortions arising from the fact that data attrition due to artifacts and behavioral false starts/non-responses yielded unequal trial numbers for each category condition (Pereira et al., 2009). For additional technical detail about the *K*-means or SVM classification procedures, see Supplementary Material: Analytical Methods – Technical Details.

This *K*-means/SVM-based procedure classified EEG trials into two classes reflecting each possible category perception. The two possible ν states and two possible μ states yielded four values for *Q(*ν*|*μ*,m)*: (1) the conditional probability *Q(*ν = *1|* μ = *1,m)* of presentation of Category 1 given the presence of the Category 1 perception-specific brain state, (2) the conditional probability *Q(*ν = *1|* μ = *2,m)* of presentation of Category 1 given the presence of the Category 2 perception-specific brain state, (3) the conditional probability *Q(*ν = *2|* μ = *1,m)* of presentation of Category 2 given the presence of the Category 1 perception-specific brain state, and (4) the conditional probability *Q(*ν = *2|* μ = *2,m)* of presentation of Category 2 given the presence of the Category 2 perception-specific brain state.

#### Computing Free Energy Differences

This final step involved entering the generative model and recognition probability distributions into the free energy Eq. 4. This yielded four brain free energy differences Δ*F(o*,μ*)* for each participant and categorization task. The four differences were also summated to yield an estimate of the total brain free energy difference for each task,

(8) $$\Delta \,F_{t\,o\,t\,a\,l}=\sum_{o}\sum_{\mu }\Delta \,F\,\left(o,\mu \right)$$

The estimates obtained via this procedure are measures of *global brain free energy differences* because scalp-recorded EEG signals index global brain activity that reflects changes in perception and cognition throughout an information-processing cycle. In the present study, this information-processing takes place across trials and the span of the entire categorization task. Thus Δ*F*_*total*_ reflects the total free energy change across a task, whereas Δ*F(o*,μ*)* reflects the free energy changes on trials where the brain’s encoding of category perceptions μ matches (*o* = μ = Category 1 or Category 2) or mismatches (*o* = Category 1, μ = Category 2; *o* = Category 2, μ = Category 1) the optimal category perceptions *o* for those trials. The free energy difference measures computed here were expressed in terms of natural units of information (nats).

#### Statistical Analysis of Brain Free Energy State Differences

To assess differences in Δ*F*_*Total*_ between tasks, a non-parametric permutation-based one-way repeated measures ANOVA was performed with a factor of Categorization Task (II, RB). All non-parametric permutation-based ANOVAs were implemented via EEGLAB. In addition, Pearson correlations *r* were used to assess the relationship between Δ*F*_*Total*_ and global WP scores; Pearson correlations were assessed via randomization testing (Efron and Tibshirani, 1993; 5000 randomizations) using custom in-house MATLAB scripts. All *post hoc* and/or multiple comparisons were corrected to control the False Discovery Rate to be less than or equal to 0.05 (Benjamini and Yekutieli, 2001); corrected *p*-values are indicated as such in the text.

#### Estimation of Free Energy Difference-Related Brain Responses

Each set of classified trials allowed the determination of associated spatiotemporal EEG patterns that characterized the large-scale neural representation μ associated with different brain free energy differences. It is of interest to characterize the spatiotemporal morphology of these EEG patterns in order to understand what visual processing stages might be related to any free energy differences observed during the present categorization task. Hence, evoked global field power (GFP) was computed by first creating event-related potential (ERP) averages of stimulus-locked EEG epochs across a participant’s 200 separate classifications at each electrode and for each free energy difference Δ*F(o*,ν*| m)*. This was achieved by separating EEG trials according to whether the K-means clustering-indexed category perception-discriminative brain states matched or mismatched the optimal category perceptions (and thus the true category labels, given optimum generative model *M*) on a given trial. Evoked GFP was computed as the standard deviation of the ERP values across electrodes for each time point (Murray et al., 2008). GFP waveforms were created separately for trials exhibiting small and large free energy differences. Grand-average waveforms were computed by averaging waveforms across participants. Statistical comparisons of GFP waveforms were computed using pointwise non-parametric randomized permutation *t*-tests (*p* < *0.05*, two-tailed, 5000 permutations) with Type-I error corrections for multiple comparisons made via a maximal statistic procedure (Nichols and Holmes, 2002). However, these statistical comparisons were used only to indicate the temporal range of GFP waveform differences and not to estimate their effect sizes, as the latter are circularly biased (Kriegeskorte et al., 2010) due to the fact that EEG trials were pre-selected on the basis of their free energy condition.

#### Resource Allocation Parameter Estimation

The resource allocation parameter ζ was estimated from each participant’s visual categorization task behavior by application of a softmax perceptual decision-making model (Reverdy and Leonard, 2016). Category perceptions were behaviorally indexed by the perceptual decision *d* made by each participant about the true stimulus category ν on a given trial. For two possible category perceptions *i* = 1,2 with equal prior probabilities *P(d_1_)* = *P(d_2_)* = 0.5,

(9) $$P\,\left(d_{1}\right)=\frac{P\,\left(d_{1}\right)\,e^{\zeta \,U\,\left(d_{1}|m\right)}}{P\,\left(d_{1}\right)\,e^{\zeta \,U\,\left(d_{1}|m\right)}+\left(1-P\,\left(d_{1}\right)\right)\,e^{\zeta \,U\,\left(d_{2}|m\right)}}=\frac{1}{1+e^{-\zeta \,\left(U\,\left(d_{1}|m\right)-U\,\left(d_{2}|m\right)\right)}}$$

where *U(d_1_|m)* and *U(d_2_|m)* are the utility functions for perceptual decisions *d*_1_ and *d*_2_, respectively, and *P(d_2_)* = *1 – P(d_1_)*. Following the definitions of the utility functions used in the derivation of Eq. 4 (see section “Free Energy Differences and Information-Processing Costs”), the utility functions were set as*:*

(10) U⁢(di|m)={ln⁡(P⁢(di|m)),for trials with decision ⁢di0,o⁢t⁢h⁢e⁢r⁢w⁢i⁢s⁢e

where

(11) $$P\,\left(d_{i}|m\right)=\sum_{j=1}^{2}P\,\left(d_{i}|\nu_{j},m\right)\,P\,\left(\nu_{j}|m\right)$$

That is, the utility for a perceptual decision is modeled as non-zero only for task trials on which that decision is made; this reflects the assumption that an observer’s perceptual decision was based on the utility of the perceived visual category. Once the utility function for a given participant’s categorization behavior data was defined, the ζ parameter for the model was estimated using logistic regression (Hosmer and Lemeshow, 2000) implemented in MATLAB. The utility of perceptual decisions did not differ across tasks or between categories within a task (see Supplementary Material: Analytical Methods – Technical Details).

Between-task differences in model parameter ζ were statistically assessed via non-parametric, permutation-based one-way repeated-measures ANOVA with a factor of Categorization Task (II, RB) implemented via EEGLAB. In addition, the Pearson correlation *r* between Δ*F*_*Total*_ and parameter ζ was calculated, with statistical significance assessed against the null hypothesis (*r* = 0) via randomization testing (Efron and Tibshirani, 1993; 5000 randomizations) using custom in-house MATLAB scripts.

## Results

All data, stimulus materials, and MATLAB data analysis scripts are available online via the Texas Data Repository at https://dataverse.tdl.org/dataverse/info_fe_eeg.

### Categorization Task Behavior and Resource Parameter Estimation

Behavior descriptive statistics are shown in Table 1. Categorization performance was above chance for both tasks: II Task, *F*(1,47) = 384.30, *p* < 0.001, η_p_^2^ = 0.89; RB Task, *F*(1,47) = 75.20, *p* < 0.001, η_p_^2^ = 0.62. Nevertheless, participants categorized the stimuli more accurately during the II Task than the RB Task, *F*(1,47) = 25.31, *p* < 0.001, η_p_^2^ = 0.35; see Table 1.

**TABLE 1:** Behavior data and fitted computational model parameters averaged across-subjects.

**Measure**	**II Task**	**RB Task**
Accuracy (%)	70 [68, 72]	63 [60, 66]
Reaction Time: Correct Trials (ms)	470 [428, 513]	507 [468, 547]
Reaction Time: Incorrect Trials (ms)	518 [468, 568]	548 [504, 592]

*95% CIs in parentheses.*

Overall response times for participants to indicate their categorization decisions were not significantly different between tasks: Categorization Task main effect, *F*(1,47) = 2.88, *p* < 0.095, η_p_^2^ = 0.06. However, across both tasks participants were faster to indicate their categorization decisions for correct versus incorrect stimulus categorizations: Categorization Accuracy main effect, *F*(1,47) = 57.25, *p* < 0.001, η_p_^2^ = 0.55; see Table 1. The Categorization Task x Accuracy interaction was not significant, *F*(1,47) = 0.52, *p* < 0.497, η_p_^2^ = 0.01.

Information processing resource parameter ζ values were negative, but these values were larger (e.g., more positive) for the RB Task (ζ = −0.73, 95% CIs [−0.93, −0.54]) versus the II Task (ζ = −1.20, 95% CIs [−1.35, −1.04]): Categorization Task main effect, *F*(1,47) = 18.53, *p* < 0.001, η_p_^2^ = 0.28.

### SVM Classifier Performance

**FIGURE 6:**
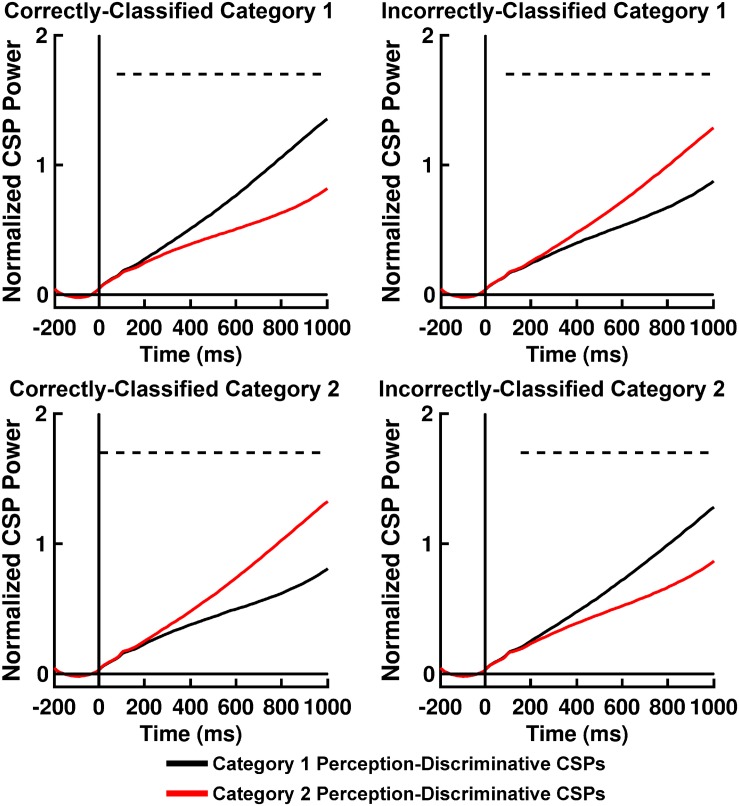
Time courses of mean II categorization task normalized perception-discriminative Common Spatial Pattern (CSP) activation power for correctly and incorrectly SVM-classified Category 1 trials (**top left** and **right panels**, respectively) and for correctly and incorrectly SVM-classified Category 2 trials (**bottom left** and **right panels**, respectively). Black lines indicate CSP component brain responses most discriminative for Category 1 perception and least discriminative for Category 2 perceptions; red lines indicate CSP component brain responses most discriminative for Category 2 perceptions and least discriminative for Category 1 perceptions. Dashed lines indicate time periods of statistically significant differences between CSP component power indicated by permutation-based statistical testing corrected for multiple comparisons across time (see section “Analytical Methods, Estimation of Free Energy Difference-Related Brain Responses”).

**FIGURE 7:**
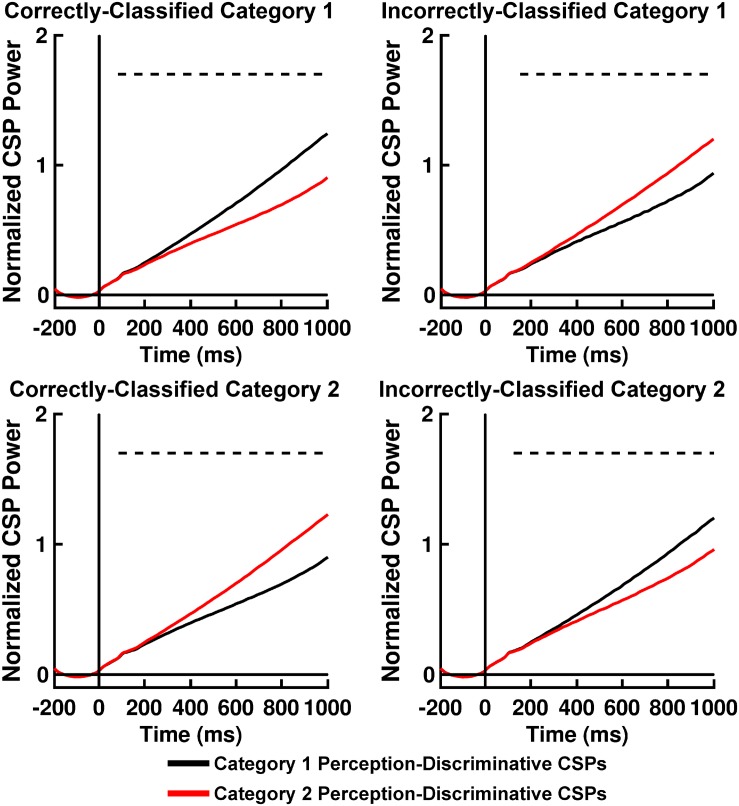
Time courses of mean RB categorization task normalized perception-discriminative Common Spatial Pattern (CSP) activation power for correctly and incorrectly SVM-classified Category 1 trials (**top left** and **right panels**, respectively) and for correctly and incorrectly SVM-classified Category 2 trials (**bottom left** and **right panels**, respectively). Black lines indicate CSP component brain responses most discriminative for Category 1 perception and least discriminative for Category 2 perceptions; red lines indicate CSP component brain responses most discriminative for Category 2 perceptions and least discriminative for Category 1 perceptions. Dashed lines indicate time periods of statistically significant differences between CSP component power indicated by permutation-based statistical testing corrected for multiple comparisons across time (see section “Analytical Methods, Estimation of Free Energy Difference-Related Brain Responses”).

Table 2 displays *K*-means accuracy, SVM accuracy, and Bayes’-optimized SVM hyperparameters grand-averaged across participants. *K*-means clustering classification accuracy was high. SVM classification accuracy for computation of *Q(*ν*|*μ*,m)* was comparable to accuracy rates for participant behavior, differing from the latter on the order of ∼ 2–3% (Table 2). The performance of this classifier can be explained by an analysis of the across-trial activation power for the perception-discriminative CSP features (Figures 6, 7). The latter showed that CSP activation power for correctly classified trials (left columns of Figures 6, 7) was greater for the CSP features corresponding to the correct versus incorrect category perception. However, CSP power for incorrectly classified trials was greater for the CSP features corresponding to the incorrect versus correct category perception (right columns of Figures 6, 7). This showed that EEG trials classified according to these CSP features tracked the category perceptions rather than the true category labels.

**TABLE 2:** Grand-average *K*-means clustering accuracy, SVM accuracy, and SVM Bayes’-optimized hyperparameters.

**Measure**	**II Task**	**RB Task**
**K-means**		
Accuracy (%)	98.3 [98.2, 98.4]	98.5 [98.4, 98.6]
**SVM**		
Accuracy (%)	66.9 [66.2, 67.6]	61.0 [60.4, 61.9]
Sigma	1871.6 [771.8, 4077.7]	3742.7 [1178.1, 7306.6]
Box	9423.1 [4065.0, 15442.8]	8219.3 [3626.8, 13997.2]

*95% CIs in parentheses. Hyperparameters are dimensionless.*

### Global Brain Free Energy Differences

Estimated global brain free energy differences are listed in Table 3. The magnitude of total global brain free energy difference Δ*F*_*Total*_ (collapsing across all possible *o* and μ states according to Eq. 8) was negatively related to the model confidence parameter ζ for both categorization tasks: II Task, *r* = −0.88, *t*(46) = −12.57, *p*_corrected_ < 0.001, two-tailed; RB Task, *r* = −0.90, *t*(46) = −14.00, *p*_corrected_ < 0.001, two-tailed. In addition, Δ*F*_*Total*_ was higher for the II Task versus the RB Task, *F*(1,47) = 18.75, *p* < 0.001, η_p_^2^ = 0.28; see Table 3.

**TABLE 3:** Estimated global brain free energy differences.

	**Δ*F*(*o* = 1,μ = 1)**	**Δ*F*(*o* = 1,μ = 2)**	**Δ*F*(*o* = 2,μ = 1)**	**Δ*F*(*o* = 2,μ = 2)**	**Δ*F*_*Total*_**
II Task	2.31 [2.20, 2.41]	4.01 [3.87, 4.14]	4.02 [3.88, 4.17]	2.29 [2.17, 2.42]	12.63 [12.59, 12.68]
RB Task	2.63 [2.52, 2.76]	3.63 [3.49, 3.76]	3.61 [3.45, 3.76]	2.66 [2.52, 2.80]	12.53 [12.40, 12.57]

*95% CIs in parentheses. Free energy differences are in units of nats.*

The FEP also makes a supplementary prediction that was tested here. According to the FEP, brain free energy minimization also minimizes the brain’s surprise and enables the brain to approach a Bayes’-optimal prediction of the causes of perceptions from the perceptions themselves, as encoded by *Q(*ν*|*μ*,m)*. Therefore, smaller brain free energy changes should occur when the brain’s representations of perceptual states approximate the category perceptions that optimally predict perceptual causes as encoded by the optimum generative model. This then suggests that global brain free energy differences Δ*F(o*,μ*)* should be smallest on trials where the brain’s encoding of category perceptions μ matches the optimal category perception *o* for those trials, whereas Δ*F(o*,μ*)* should be largest when the brain’s perceptual encoding and the optimal category perception mismatch. In order to test this prediction, individual free energy states were first collapsed to yield average free energy values for mismatching and matching *o* and μ states. Then a non-parametric permutation-based two-way repeated measures ANOVA was performed on the collapsed data, with factors of Categorization Task and Free Energy Difference State (Matched, Mismatched). The ANOVA confirmed this prediction; Free Energy Difference State main effect, *F*(1,47) = 154.74, *p* < 0.001, η_p_^2^ = 0.77; see Table 3. Moreover, the Categorization Task main effect was also significant, *F*(1,47) = 18.75, *p* < 0.001, η_p_^2^ = 0.28, in accordance with the between-task analysis of Δ*F*_*Total*_ reported above. Furthermore, a significant Categorization Task × Free Energy Difference State interaction indicated that the magnitude levels of these free energy states were different across categorization tasks, *F*(1,47) = 23.28, p < 0.001, η_p_^2^ = 0.33. Follow-up analyses revealed that free energy differences for mismatching *o* and μ states were higher for the II versus RB task, *F*(1,47) = 23.17, *p*_corrected_ < 0.001, η_p_^2^ = 0.33, whereas free energy differences for matching *o* and μ states were lower for the II versus RB tasks, *F*(1,47) = 23.37, *p*_corrected_ < 0.001, η_p_^2^ = 0.33; see Table 3.

### Free Energy Difference-Related Brain Responses

**FIGURE 8:**
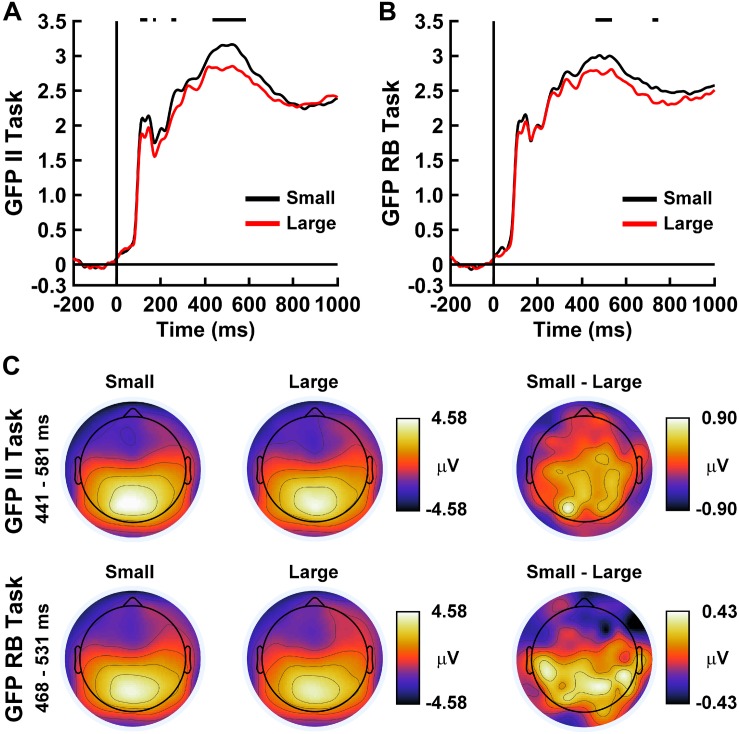
Time courses of ERP global field power (GFP) for **(A)** the II Task and **(B)** RB Task for small free energy differences (black lines) and large free energy differences (red lines). GFP values are in μV. Horizontal black lines indicate time ranges demonstrating significant between-condition differences between GFP waveforms as indicated by permutation-based statistical testing corrected for multiple comparisons across time (see section “Analytical Methods” and “Estimation of Free Energy-Related Brain Responses”). **(C)** Topographical head maps show mean ERP values across the scalp for the indicated free energy differences and between-condition contrasts averaged over the indicated time ranges. The noses of the headmaps point upward; light/dark colors indicate ± values.

Figures 8A,B displays the grand-average evoked GFP of the ERP correlates of brain states corresponding to small and large brain free energy differences for the II and RB categorization tasks. For both categorization tasks, GFP waveform differences were present primarily during intermediate stages of visual processing (II Task: 441–581 ms post-stimulus onset; RB Task: 468–531 ms post-stimulus onset). Topographical mapping (Figure 8C) illustrated that these GFP differences were associated with a difference in evoked responses over posterior and central scalp sites.

### Mental Workload and Brain Free Energy

Subjective ratings of mental workload (as indexed via the WP questionnaire) were slightly larger for the RB task (0.50, 95% CIs [0.45, 0.55]) than the II task (0.48, 95% CIs [0.43, 0.54]), but this difference only reached trend-level statistical significance, Categorization Task main effect, *F*(1,47) = 2.94, *p* < 0.094, η_p_^2^ = 0.06. However, global WP scores were significantly negatively correlated with Δ*F*_*Total*_ in the RB task but not the II task: RB Task, *r* = −0.39, *t*(46) = −2.59, *p*_corrected_ < 0.009, two-tailed; II Task, *r* = −0.22, *t*(46) = −1.52, *p*_corrected_ < 0.200, two-tailed. Also, global WP scores were significantly positively correlated with model confidence parameter ζ for the RB task but not the II task: RB Task, *r* = 0.35, *t*(46) = 2.52, *p*_corrected_ < 0.014, two-tailed; II Task, *r* = 0.19, *t*(46) = 1.32, *p*_corrected_ < 0.188, two-tailed.

## Discussion

The present study tested the theoretical relationship between information-processing resource costs allocated through mental effort and an information-theoretic property of the brain called free energy. This was accomplished by quantifying the free energy differences of global brain states from participant behavior and EEG responses elicited during a simple visual categorization task. Information-processing resource costs were estimated via computational modeling of categorization behavior, whereas the subjective expression of mental effort was indexed via participant ratings of mental workload. The present findings support four theoretical predictions for the relationship of brain free energy to neurocognitive information-processing resource costs and mental effort (see section “Introduction”). To the present author’s knowledge, this study is the first empirical assessment of the relationship between mental effort, brain free energy, and neurocognitive information-processing.

### Relationship of Brain Free Energy to Neurocognitive Information-Processing Costs

The first prediction tested by the present study was that brain free energy differences would negatively correlate with information-processing resource parameter ζ. This prediction was based on thermodynamical approaches to bounded rational decision making (Ortega and Braun, 2013). Here, the ζ parameter reflects the information-processing resource costs of a decision maker by acting as an “inverse temperature” parameter for the probability distributions that describe the decision maker’s information-processing changes (although the same prediction can also been reached via considerations of active inference; Friston et al., 2016). This prediction was confirmed for both the information-integration (II) and rule-based (RB) categorization tasks. Across-participants, as total global free energy difference Δ*F*_*Total*_ decreased in magnitude, the ζ parameter increased in magnitude.

The second prediction tested by the present study was that brain free energy differences would be smaller, and parameter ζ would be larger, for the visual categorization task that theoretically required a larger versus smaller amount of information-processing resource costs. The present RB task theoretically required a larger amount of information-processing resource costs than the II task. This is because the category space inferred by the participants in the RB task imposed a larger degree of interference-related representational capacity constraints among similar task-related neurocognitive representations (Shenhav et al., 2017) that required the allocation of additional information-processing resources to resolve. The RB task utilized visual categories with highly complex visual feature overlap and required categorization based on verbalizable rules known to be mediated by an explicit representational system that requires effortful attention for its operation (Maddox and Ashby, 2004). For the II task, the visual feature overlap was less complex and categorization was based on non-verbalizable criteria mediated by an implicit system that operates automatically. This prediction was confirmed in that the total brain free energy difference Δ*F*_*Total*_ was smaller, and parameter ζ larger (more positive), for the RB versus II task. (The resource parameter differences were not due to differences in the utility of perceptual decisions across tasks or between categories within a task; see Supplementary Material: Analytical Methods – Technical Details.) Future research could investigate how information-processing resource allocation is reflected in parameter ζ across category spaces that realize a wider and more fine-grained range of representational capacity constraints than used here. These spaces could be formed by crossing the two types of category representation with simple and complex patterns of visual feature overlap over single and multiple feature dimensions.

### Relationship of Brain Free Energy to Subjective Ratings of Mental Effort

Global brain free energy and the ζ parameter were also related to participant ratings of the subjective expression of mental effort (Shenhav et al., 2017) as indexed by participant ratings of mental workload. It was predicted that to the extent that mental effort reflects the allocation of information-processing resources, ratings of mental workload should positively correlate with the ζ parameter values and negatively correlate with global brain free energy. These two predictions were predicated on the finding that people subjectively experience mental effort as psychological “work” in proportion to the actual effort with which they engage in a task (Kantowitz, 1987). These predictions were confirmed for the RB task but not the II task. One possible reason for this may be that in the RB task participants were more subjectively sensitive to the allocation of information-processing resources. The larger ζ parameter for the RB task suggests a high degree of resource allocation that may have been more greatly reflected in an individual’s subjective experience of their mental effort. A second possibility is that the present implementation of the WP questionnaire used to index mental workload was insufficiently sensitive to capture a subtler relationship between experienced effort and information-processing allocation during the II task (see section “Experimental Methods, Subjective Assessment of Mental Workload,” and Supplementary Material: Subjective Assessment of Mental Workload via the Workload Profile for a description of the WP questionnaire). Participants were instructed to give their ratings within a particular range, but the questionnaire used here did not provide a visual scale on which the basis of such ratings could be made. It is possible that use of a visual scale might yield more accurate and fine-grained subjective estimations of workload that in turn would more robustly correlate with the ζ parameter. Also participants were instructed to evaluate their workload for each task independently, but it is possible that in doing so participants did not adequately base their subjective estimations for each task relative to a common experienced baseline of mental effort. One way to address this might be to change the instructions such that participants rated their experience of mental work in one task relative to the work they experienced in the other task, rather than rating each task independently.

### FEP-Based Expected Patterns of Brain Free Energy Differences

A supplementary prediction made by the FEP is that smaller brain free energy differences should be accompanied by a higher probability that the brain’s representations of perceptual states approximate the category perceptions that optimally predict perceptual causes as encoded by the optimum generative model. This prediction was fully supported by the present data for both categorization tasks (see section “Results, Global Brain Free Energy Differences”). Global brain free energy differences were smallest over trials where the brain’s encoding of category perceptions μ matched the optimal category perception *o* for those trials and were largest for trials where the brain’s perceptual encoding and the optimal category perception mismatched. These free energy differences were characterized by different levels of EEG global field power that was maximal over posterior and central scalp regions during intermediate to late stages of visual processing (Figure 8). Moreover, these small/large free energy differences roughly corresponded to trials that were correctly and incorrectly discriminated by the brain, respectively. These findings support the theoretical claim that minimization of brain free energy indirectly minimizes the brain’s surprise about its categorizations and enables the brain to approach Bayes’-optimal representation and prediction of the conceptual labels of the categories.

One question raised by these findings is how to reconcile the interpretation of brain free energy minimization of surprise with the bounded rationality-based interpretation that brain free energy minimization reflects changes in the costs of mental information processing. Answering this question is outside the scope of the present paper, but one hypothesis is that the successful minimization of surprise requires additional mental information processing resources than when surprise is not minimized or minimized to a lesser degree. This would then suggest that a greater degree of mental effort and associated information processing corresponds to an increased likelihood of accurate stimulus processing. This possibility is consistent with the present observations; Δ*F(o*,μ*)* was smaller for matching *(o*,μ*)* states during the task with the higher (II task) versus lower (RB task) overall accuracy. However, Δ*F(o*,μ*)* was higher for mismatching *(o*,μ*)* states during the II versus RB task, suggesting that the larger Δ*F*_*Total*_ found for the II task versus RB task arises from a larger contribution to the total free energy difference from incorrect trials for the II task.

This latter finding raises a difficulty for theories of brain free energy. The minimization of brain free energy also minimizes the brain’s surprise by increasing the precision of its neural representations (Friston, 2010; Friston et al., 2015, 2016). Yet of the two categorization tasks utilized in the present study, the task with the greater free energy difference had the greater performance accuracy on average. One possible explanation for this may be that optimum behavioral performance does not result from completely precise brain representations, but instead requires some degree of neural variability in order to engage flexible neurocognitive information processing (Garrett et al., 2011). This is consistent with evidence that the brain exhibits the property of criticality – an optimal balance between ordered and disordered states (Beggs, 2008; Shew and Plenz, 2013; Hesse and Gross, 2014; Atasoy et al., 2017). Investigation of the connection between brain free energy and criticality is a topic for future research.

### Validity of Resource Allocation Parameter Estimation Method

In the present study, global brain free energy differences were computed from a definition of free energy difference (Eq. 4) that allows for general statistical distributions estimated from the data, but with the ζ parameter an unknown variable implicit within these distributions. It was hypothesized that these empirically estimated distributions would behave as if governed by an “inverse temperature” parameter and this would then be reflected in the observed relationship of free energy to the ζ parameter as estimated via an additional method. Here the ζ parameter was estimated from each participant’s visual categorization behavior by application of a softmax perceptual decision-making model (Reverdy and Leonard, 2016) with a mathematical form that minimizes the free energy difference of a bounded-rational decision maker (Ortega and Braun, 2013). Estimating the ζ parameter directly from behavioral data rather than brain data avoids any possible statistical circularity that may arise when relating the parameter estimates to free energy. However, this model depended on the computation of utility functions for a participant’s perceptual decisions, so the validity of interpreting ζ in terms of information processing resources depends on these utility functions not differing between categorization tasks or across perceptual category decisions. (Note also that free energy can reflect changes in utility too). Fortunately, this was the case (see Supplementary Material: Analytical Methods – Technical Details), reflecting the fact that utility was held relatively constant in the present study; participant performance was only rewarded in terms of a fixed amount of course credit or monetary payment. Utility differences across participants were likely due to idiosyncratic motivations on the part of the participants to perform well and reduce negative performance feedback.

Another issue with the present resource allocation parameter estimation procedure is the interpretation of the negative sign of the ζ values; according to the thermodynamic approach to decision making, negative ζ values are interpreted as indicating pessimistic decision makers who are “anti-rational” (Ortega and Braun, 2013). It is unclear if this interpretation is applicable to the present sample of participants. Future studies could address this issue by recording participant attitudes toward the task via questionnaire.

Finally, this parameter estimation procedure was based on perceptual decisions indicated by overt behavioral responses. Perceptual observations are imperfectly indexed via behavior. There may be cases where a participant experiences a certain category perception but makes an opposite decision or behavioral response due to internal noise. This limitation might be improved by better training of the participants on the structure of the category space and/or on the overall task procedure.

### Validity of Brain Free Energy Quantification Procedure

An important remaining question to address here is if the brain free energy difference quantification procedure introduced in this study validly indexes brain free energy at all. There are several issues to consider. The first is the method used to estimate the optimum generative model distribution *P(o*,ν*|M)*. Here a generative model was used that assumed an optimal categorizer with perfect knowledge of the category structure of the perceptual space, i.e., perfect knowledge of how the perceptual similarity among stimuli maps to the category labels. Such knowledge is possible in principle with the category spaces used in this study (Figure 3), as specific ranges of stimulus spatial frequency and orientation combinations had one-to-one mappings to the category labels; it was never the case that these specific feature combinations mapped with some probability to both categories. The generative model also assumed an optimal perceiver who could perceptually discriminate between all the different possible spatial frequencies and orientations of the stimuli. Thus in a sense, the free energy measure used here indexes a participant’s departure from perfect categorization and category perception performance, but this indexing is made on the basis of brain states rather than behavior. Nevertheless, it would be instructive to perform brain free energy quantification using realistic generative models that accounted for how the stimulus features were jointly distributed across the category space, as well as accounting for decrements in learning the category space, decrements in perceiving the perceptual distinctions among stimulus features, or both. For example, category learning can be modeled as a process in which the brain learns to partition a stimulus space into regions of perceptually similar stimuli and assign category labels to those regions on the basis of the distance of a stimulus to a decision boundary in the category space (Ashby and Maddox, 1993; Nomura and Reber, 2012). Alternatively, categorization could be modeled using abstract Markov decision processes implemented within an active inference framework (Schwartenbeck and Friston, 2016). A third option would be to empirically estimate the brain’s generative model by using machine learning classification of brain responses to compute the empirical likelihood distribution *P(o|*ν*,m)* and the posterior *P(*ν*| m)* such that *P(o*,ν*| m)* = *P(o|*ν*,m)P(*ν*| m)* under the assumption that this estimate reflects the true frequencies of co-occurrence of *o* and ν created by the brain’s generative model (subject to some measurement noise). How different methods of generative model estimation affect free energy quantification is an important topic for future research.

A second issue regarding the validity of the present free energy measure was the use of category perception-discriminative brain states to estimate the neural representations μ that parameterize the recognition distribution *Q(*ν*|*μ*,m)*. This choice was based on the reasoning that when free energy is minimized, *Q(*ν*|*μ*,m)* ≈ *P(*ν*|o,M)*. Thus if the brains of the participants minimized free energy during the categorization tasks, then the information encoded by neural state μ should approximately reflect the information encoded by their brains about the observations *o*. In other words, the recognition distribution was estimated here by the empirical posterior mapping between the brain’s perceptual encodings and the category labels ν. One concern with this approach is that the distributions estimated in this manner clearly deviate from the optimum posterior distribution. This is not problematic, however, because this information is precisely what the free energy measure is supposed to quantify, i.e., the accuracy of the brain’s encoding of the true posterior distribution. Another concern with this approach is how accurately the brain states used to estimate the recognition distribution encoded the category perceptions *o*. These brain states were identified using an EEG feature extraction method (Koles, 1991; Müller-Gerking et al., 1999; Ramoser et al., 2000) (see section “Analytical Methods, Global Brain Free Energy Difference Quantification”) that maximally discriminated between the two possible category perceptions as indicated behaviorally by a participant. Thus, technically, the brain states identified using this procedure encoded as much information as possible about a participant’s perceptual decisions. Nevertheless, using participant behavior to define the brain states was necessary, as no other method other than behavioral report is available to index an individual’s subjective conscious perceptions (Farthing, 1992). Hence, to the extent that these decisions were directly based on the participant’s category perceptions, then the assumption that these brain states encode information specific to each category perception is reasonable. While it is likely that these brain states also encode decision-making and motor response processes that are unrelated to perception *per se*, the presence of such information in the μ state estimate would only be problematic if these latter processes differentiated between categories. This is unlikely, however, as no significant accuracy or response time differences were observed between categories for either categorization task (see Supplementary Material: Auxiliary Behavior Analysis – Between-Category Comparisons). Although it is possible that the extra information encoded in μ might have acted as noise that reduced the accuracy of the *Q(*ν| μ,*m*) estimate, free energy differences were still observed between matching/mismatching *o* and μ states and free energy still correlated with the ζ parameter of both task and mental workload estimates of the RB task. Thus the present findings are conservative estimates of these quantities and correlations.

A third issue regarding the validity of the present free energy measure is the degree to which the measure is dependent on the quality or performance of the classifiers used to estimate *Q(*ν*|*μ,*m)* from the EEG data. This issue is analyzed in depth in the Supplementary Material (see section “Influence of Classifier Performance on Free Energy Estimation”). Here it was shown that a good classifier will yield an accurate free energy measure that reflects the brain’s stimulus encoding and discrimination capabilities, whereas a poor classifier will fail to reflect these capabilities and thus decrease the sensitivity of the free energy measure. Nevertheless, if free energy differences are still observed in the latter case, then such findings may be considered to be conservative measurements of brain free energy. The analysis presented in the Supplementary Material shows that the classifiers used in the present study were as high-performing as possible. Classification was based on maximally informative CSP-extracted EEG features that were discriminative for the brain’s encoding of its category perceptions. Accuracy rates of the K-mean classifiers were high, whereas the accuracy rates of the SVM classifiers used to estimate *Q(*ν*|*μ*,m)* were comparable to the observed categorization task accuracy rates. Moreover, a direct comparison of brain free energy computed using the classifier estimate of *Q(*ν*|*μ*,m)* to free energy computed using a recognition distribution estimate calculated directly from behavioral categorization performance showed that the present classifiers were sufficiently sensitive to probe the statistics of the relevant brain states (see Supplementary Material: Influence of Classifier Performance on Free Energy Estimation). Nevertheless, an important topic for future research is to determine if other classifier algorithms and/or classification procedures will yield more accurate estimates of *Q(*ν*|*μ*,m)* and brain free energy.

An additional point to note is that the use of a classifier-based brain free energy estimator avoided any potential statistical circularity that may arise when relating the ζ parameter estimates to brain free energy when both are estimated directly from behavioral data. Participant responses were used to separate EEG trials into one of two groups associated with a specific category perception; these trial groups were then entered into the EEG feature extraction procedure used to identify the category perception-discriminative brain states. However, participant behavior was not used for the actual trial classification that produced the estimates of *Q(*ν*|*μ*,m)*. Thus the present free energy measure is derived directly from brain activity. This argues for its interpretation as reflecting an actual, physical property of the brain, rather than a useful computational descriptor of the brain’s dynamics. The viewpoint espoused here is that brain free energy does not directly correspond to the brain’s energetic capacity to perform work, but does reflect information states of the brain that are in fact physical (Street, 2016). Specifically, free energy is an information-theoretic system property that reflects neurocognitive information processing among the widespread brain networks representing the brain’s perceptual and conceptual states.

A fourth issue regarding the validity of the present free energy measure is the appropriateness of using EEG as a method to index the brain responses associated with brain’s generative model and free energy. There is a great deal of theoretical work describing the brain’s generative model and its approximately Bayesian processing in terms of the spatiotemporal activity of neuronal networks across the different levels of the brain’s recurrent neural hierarchy (Zeki and Shipp, 1988; Felleman and Van Essen, 1991). This theoretical framework is called *predictive coding* and it has substantial empirical support (Murray et al., 2002; Summerfield et al., 2008; Garrido et al., 2009; Egner et al., 2010; Kok and De Lange, 2015; Aitchison and Lengyel, 2017). In this framework, higher levels of the neural hierarchy predict feedforward input from lower levels, which reflect the conditional expectations of signals from even lower levels. Sensory signals are encoded at the lowest levels of the hierarchy and represent conditional expectations of external world input. These expectations are compared with top-down predictions signaled from the higher representational levels via feedback connections. Any resulting prediction error is passed forward to the high-level networks, which optimize their predictions so as to reduce prediction error at the lower levels. The process cycles until prediction error is minimized and conditional expectations are maximized at all representational levels. Under certain assumptions about how the neural representations of the generative model are encoded (i.e., Gaussian statistical distributions, free energy linearization via Laplacian approximation), free energy corresponds to the difference between an internal model’s predictions and the to-be-predicted neural representations (Friston, 2010; Gershman, 2019). Free energy minimization is then equivalent to explaining away prediction errors, which can be realized neurophysiologically in terms of top-down inhibition of bottom-up excitatory inputs at lower hierarchical levels (Mumford, 1992; Friston, 2008). Hence, free energy minimization optimizes empirical priors (the probability of causes at a specific level, given causes in the preceding level) at all levels of the neural hierarchy, providing a mechanism for the formation of prior beliefs (Lee and Mumford, 2003; Kersten et al., 2004; Friston, 2010).

Importantly for the present study, scalp-recorded EEG methods detect neuronal signals emanating from superficial and deep cortex (Cuffin and Cohen, 1979; Mosher et al., 1993; Nunez and Srinivasan, 2006; Tenke and Kayser, 2015), regions that contain neurons corresponding to bottom-up error processing units and top-down predictive units, respectively (Friston et al., 2017). Scalp EEG can also detect neuronal signals originating from low and high level visual cortex, which putatively reflect neural representations of category perceptions and their labels (Hochstein and Ahissar, 2002; Nomura et al., 2007; Wang et al., 2010). This supports the use of EEG to index the brain states encoding *Q(*ν*|*μ*,m)*. However, scalp-level EEG signals reflect a simultaneous mixture of all of this cortical activity due to volume-conduction of bioelectric cortical signals as they travel through the head from the cortex to the scalp (Nunez and Srinivasan, 2006). Thus the validity of the present method depends on its ability to separate these mixed cortical signals at the level of the scalp rather than indexing this information at the level of localized neural sources.

This signal-separation was achieved using machine learning classifiers, which identified the presence of state-specific information in the EEG signals. The K-means clustering and SVM classifiers used to compute *Q(*ν*|*μ*,m)* were trained on EEG features that maximally discriminated between the two possible category perceptions. Thus these classifiers should have been maximally sensitive to the portions of the EEG signals that reflected the brain’s representation of μ. This conclusion is supported by the very high classification accuracy observed for the *K*-means clustering classifier, the similarity in classification accuracy between the SVM classifier and participant categorization task accuracy, and by the analysis of the activation power for the normalized CSP features (Figures 6, 7). The latter showed that EEG trials classified according to these features tracked the category perceptions rather than the true category labels. This suggests that the feature extraction procedure successfully partitioned information in the EEG signals related to the brain’s representation of the category perceptions.

Thus the present findings support the use of machine learning classification as an objective way to determine the trial-by-trial presence of category perception-specific brain states for the computation of Δ*F(o*,μ*)*, even when applied to brain state measures that have poor spatial sampling such as scalp–recorded EEG. Nevertheless, future research could improve upon this method by using brain recording methods with better spatial resolution than scalp EEG, such as functional magnetic resonance imaging (fMRI) or intracranial EEG recordings. It should be noted, however, that the SVM classifiers required clearly defined task conditions in order to characterize *Q(*ν*|*μ*,m)*. Future research needs to develop new ways to extend this free energy quantification procedure to brain resting state measurements or tasks (e.g., mental arithmetic, motor grasping, vigilant attention tasks) that engage ongoing brain activity without behavioral responses tied to specific external events.

## Conclusion

In conclusion, this study tested predictions originating in the thermodynamical approach to bounded rational decision making concerning the relationship between mental effort, information-resource processing costs, and brain free energy. Brain free energy differences negatively correlated with the increased allocation of information-processing resources and were smaller for a visual categorization task that required expenditure of a larger versus smaller amount of information-processing resource costs. Ratings of mental workload were positively correlated with the level of information-processing resource costs, and negatively correlated with global brain free energy difference, only for the categorization task requiring the larger resource costs. These findings provide the first empirical evidence of a relationship between mental effort, brain free energy, and neurocognitive information-processing.

## Data Availability Statement

The datasets generated for this study are available on request to the corresponding author, or may be downloaded from the Texas Data Repository at https://dataverse.tdl.org/dataverse/info_fe_eeg.

## Ethics Statement

This study was carried out in accordance with the recommendations of the Institutional Review Board at Texas State University with written informed consent from all participants. All participants gave written informed consent in accordance with the Declaration of Helsinki. The protocol was approved by the Institutional Review Board at Texas State University.

## Author Contributions

LT contributed to the experimental design, data collection and analysis, and manuscript preparation.

## Conflict of Interest

The author declares that the research was conducted in the absence of any commercial or financial relationships that could be construed as a potential conflict of interest.
