# Supplementary material for: Mental Effort and Information-Processing Costs Are Inversely Related to Global Brain Free Energy During Visual Categorization
Source: Front Neurosci. 2019 Dec 5;13:1292. doi: 10.3389/fnins.2019.01292 (PMC6906157; doi:10.3389/fnins.2019.01292)
Supplement: Supplementary file 1 [file Data_Sheet_1.pdf]

Supplementary Material

Derivation of Free Energy Bound on Surprise

An influential theory of how the brain predicts observations – *the Bayesian brain hypothesis* (Lee and Mumford, 2003; Knill and Pouget, 2004; Doya et al., 2007) – considers variables drawn from a *prior distribution P(v|m)* that specifies the different hidden states of the environment. These hidden states are encoded by the brain, but this process is usually noisy, incomplete, and/or ambiguous to the point that different hidden states can credibly produce the same (or highly similar) observations. Hence observations created by the brain's internal "generative" model are drawn from a *likelihood distribution P(o|v,m)* that is conditional on the neural representations of the hidden variables; this distribution specifies the probability of obtaining an observation given hypothetical values of the hidden states. Together the prior and the likelihood distributions compose the brain's *generative model*, *P(o,v|m) = P(o|v,m)P(v|m)*, which is transformed via Baye's rule to yield a *posterior distribution P(v|o,m)* indexing the probability that the brain represents certain values of the hidden states given its observations:

$$P(v \mid o,m) = \frac{P(o \mid v,m)P(v \mid m)}{P(o \mid m)} \quad , \qquad \text{(S1)}$$

where

$$P(o \mid m) = \sum_{v'} P(o \mid v',m)P(v' \mid m) \quad . \qquad \text{(S2)}$$

The distribution *P(v|o,m)* represents the brain's posterior "belief" about the hidden state of the environment given generative model *m*, whereas the *marginal likelihood distribution P(o|m)* quantifies the *model evidence* for *m*. The brain can realize different generative models of different quality. In the case where the brain encodes a theoretically-best possible (i.e. "correct" or "true") generative model – that is, a model that reflects a true posterior distribution *P(v|o,M)* – we denote this optimum generative model by *M*.

According to the Bayesian brain hypothesis, Bayesian agents transform prior beliefs into posterior beliefs according to Bayes' rule. However, in many situations, a direct computation of the true posterior *P(v|o,M)* is computationally-intractable because the causes of observations are hidden and the number of possible causes of observations can be very large (Dayan et al., 1995; Pio-Lopez et al., 2016). The FEP approach circumvents this by assuming that the brain performs approximate Bayesian inference in which an optimal distribution *Q(v|μ,m)* is estimated that is as close as possible to *P(v|o,M)*. This distribution is induced by the brain's generative model "over the variables that parameterize the evidence or marginal likelihood of external states" (Friston, 2012, p. 2109); these variable parameters are represented by internal neural states μ. In other words, *Q(v|μ,m)* is a fictional distribution putatively created by the brain according to its internal model to estimate how the hidden causes of observations may be predicted from observations. The estimation of *Q(v|μ,m)* is achieved by minimizing the brain's surprise about its observations. The brain's surprise is given as the negative log-evidence for model *m* (Pio-Lopez et al., 2016; Gershman, 2019),

$$S = -\ln P(o \mid m) = F(o,\mu) - D_{KL}[Q(v \mid \mu,m) \,||\, P(v \mid o,M)], \qquad \text{(S3)}$$

where $D_{KL}[\cdot||\cdot]$ denotes the Kullback-Leibler (KL) divergence between two distributions and $F(o,\mu|m)$ is the *variational free energy*,

$$F(o,\mu) = \sum_{v} Q(v \mid \mu,m) \ln Q(v \mid \mu,m) - \sum_{v} Q(v \mid \mu,m) \ln(P(o,v \mid M)). \qquad \text{(S4)}$$

Free energy is measured in units of information that depend on the base of the logarithms (here, in natural logarithmic units or nats).

The KL-divergence in Equation S3 is a measure of the distance between two probability distributions and is always non-negative; it equals 0 when $Q(v|\mu,m) = P(v|o,M)$. The brain cannot directly minimize the KL-divergence as a function of $Q(v|\mu,m)$ because this term also includes $P(v|o,M)$. However, the brain can tractably minimize the free energy $F(o,\mu)$ because that term only requires knowledge of $P(o|v,M)$ and $P(v|M)$, which the brain can estimate from its generative model (Pio-Lopez et al., 2016). Furthermore, minimizing the free energy is equivalent to minimizing the KL-divergence, as the two terms must balance each other out given that surprise as described by Equation S3 is fixed as a function of $Q(v|\mu,m)$ (Pio-Lopez et al., 2016; Gershman, 2019):

$$\left.\begin{aligned} F(o,\mu) &= D_{KL}[Q(v \mid \mu,m) \,||\, P(v \mid o,M)] - \ln P(o \mid m) \\ &\geq -\ln P(o|m) \end{aligned}\right\} . \qquad \text{(S5)}$$

Thus by estimating a distribution $Q(v|\mu,m)$ that minimizes free energy, the brain indirectly computes an optimal estimate of the true posterior distribution $P(v|o,M)$ and attains an approximately Baye's-optimal belief about the hidden states of the environment given the brain's observations and generative model. Moreover, it is also clear from Equation S5 that the free energy provides an upper bound on surprise, so minimizing free energy is also equivalent to minimizing the brain's surprise about its observations. This is the essence of the FEP for the brain.

Free Energy Differences Under Constant Prior

Consider main text Equation 3 and assume that an individual's decision processes follow general statistical distributions with an implicit resource parameter ζ. Identify a decision maker's initial information state $p_0(v|m)$ with their prior beliefs $P_0(v|m)$ and their final information state $q(v|m)$ with the recognition distribution $Q(v|\mu,m) \approx p(v|o,m)$, with both distributions entailed by the decision maker's generative model m. Define the decision's utility as $U(o,v|M) = ln(P(o|v,M)) = ln(P(o,v|M)/ P(v|M))$, which is entailed by the optimum generative model M. Then (approximate) Bayesian inference satisfies a variational principle in the free energy as follows (see discussion section of Ortega and Braun, 2013):

$$\begin{aligned} \Delta F(o,\mu) &= \sum_{v} q(v \mid m) \ln \frac{q(v \mid m)}{p_0(v \mid m)} - \sum_{v} q(v \mid m) U(o,v \mid M) \\ &= \sum_{v} Q(v \mid \mu,m) \ln \frac{Q(v \mid \mu,m)}{P_0(v \mid m)} - \sum_{v} Q(v \mid \mu,m) \ln P(o \mid v,M) \quad , \\ &= \sum_{v} Q(v \mid \mu,m) \ln \frac{Q(v \mid \mu,m)}{P_0(v \mid m)} - \sum_{v} Q(v \mid \mu,m) \ln \frac{P(o,v \mid M)}{P(v \mid M)} \end{aligned} \qquad \text{(S6)}$$

where $P(o,v|M) = P(o|v,M)P(v|M)$. When the true prior distribution is known by the decision maker and does not change over an information-processing cycle, $P_0(v|m) = P_0(v|M) = P(v|M)$. This was

the case in the present study because 1) participants were told that they would be presented with equal numbers of stimuli from each category, and 2) the probability of a given stimulus category remained constant across each categorization task; thus they should not have been inclined to alter their internal estimation of the prior distribution during task performance. Under these conditions,

$$\begin{aligned}\Delta F(o,\mu) &= \sum_{\nu} Q(\nu \mid \mu,m)\ln\frac{Q(\nu \mid \mu,m)}{P_0(\nu \mid M)} - \sum_{\nu} Q(\nu \mid \mu,m)\ln\frac{P(o,\nu \mid M)}{P_0(\nu \mid M)} \\ &= \sum_{\nu} Q(\nu \mid \mu,m)\ln Q(\nu \mid \mu,m) - \sum_{\nu} Q(\nu \mid \mu,m)\ln P(o,\nu \mid M) \ , \\ &\quad + \sum_{\nu} Q(\nu \mid \mu,m)[\ln P_0(\nu \mid M) - \ln P_0(\nu \mid M)]\end{aligned} \qquad \text{(S7)}$$

which then yields the final form of the free energy difference:

$$\Delta F(o,\mu) = \sum_{\nu} Q(\nu \mid \mu,m)\ln Q(\nu \mid \mu,m) - \sum_{\nu} Q(\nu \mid \mu,m)\ln P(o,\nu \mid M)\,. \qquad \text{(S8)}$$

The final form of this expression is equivalent to Equation S4 and main text Equation 1. This equivalence illustrates that, in the case of a known constant prior, absolute free energy levels may also be considered to be free energy differences relative to a zero baseline. The free energy difference expressed by Equation S8 is always greater than or equal to the brain's surprise and thus is always non-negative in value (see Supplementary Material: Derivation of Free Energy Bound on Surprise section, above).

Experimental Methods – Technical Details

Categorization Task. Participants were assigned to one of four different versions of the II and RB Tasks, where each version differed in terms of the particular combinations of spatial frequency and orientation that defined the categories, while maintaining the basic category structure illustrated in main text Figure 3. The different task versions were counterbalanced across-participants, which enabled the key visual features of the stimuli (mean luminance, contrast, and spatial frequency) to be approximately matched across categories for each participant in the RB task and matched for each category across participants in the II task. The matching of stimulus features in this manner minimized the possibility that any observed differences in the electrophysiological brain responses were due to differences in the physical image properties of the stimuli. Sine-wave gratings spanned ~ 4.2° of visual angle at a viewing distance of 75 cm. Spatial frequencies ranged from 1.19 to 4.29 cycles per degree in 16 equally-spaced values; orientations ranged from 10 to 80 degrees from horizontal in 40 equally-spaced values. Stimuli were uniformly sampled (with replacement) from the stimulus space. Participants categorized 448 sine-wave gratings presented in four blocks during each task session (112 stimuli per block, 56 stimuli per category per block).

EEG Pre-Processing. The continuous EEG signals were divided into 2 second epochs time-locked to stimulus onset, transformed to an average reference, and band-pass filtered between 0.1 and 30 Hz (EEGLAB-based 8449 point zero phase shift sinc FIR filter with 0.1 Hz transition bands and 0.05 Hz and 30.05 Hz -6 dB cutoff frequencies; filter edge effects were reduced via zero-padding and use of a Hamming window). Epochs were then truncated to −200 ms before to 1000 ms after stimulus onsets, and baseline corrected to the −200 to 0 ms pre-stimulus interval. Next muscle and signal artifacts were removed from the EEG record by visual inspection. Bad EEG channels were replaced using an EEGLAB-based spherical spline interpolation algorithm (Perrin et al., 1987; m = 50, 50 term expansion) applied to the remaining channels. The mean number of interpolated channels was 1.5 ± 0.2 (approximately 2.0% of all channels). Categorization task trials with RTs < 100 ms and

> 2000 ms were excluded from further analysis. The lower bound criterion minimized contamination of the EEG response with motor processes related to the indication of a categorization decision via button press; the upper bound criterion reflected the time limit to respond in the categorization task.

Blink and saccade-related electroocular (EOG) artifacts were removed by first computing two EOG channels: one formed from the bipolar montage of site NZ and the average of the two electrodes located at the inferior orbits of the eyes (sensitive to blinks and vertical saccades) and a second formed from the bipolar montage of AF9 and AF10 (sensitive to horizontal saccades). Then EEG trials containing EOG amplitudes higher than 50 µV or lower than −50 µV (after removal of the constant direct current offset from the EOG signals) were rejected from the analysis in MATLAB via automatic algorithm. These rejection criteria were applied over the −200 pre-stimulus to 1000 ms post-stimulus interval. Then, a second round of manual artifact scoring was performed because ocular artifact correction algorithms occasionally fail to remove all ocular artifacts on some trials. The derived horizontal and vertical EOG channels were removed from the data after elimination of EOG artifacts. On average, 211 ± 8 correct and 125 ± 6 incorrect trials remained for the RB task and 237 ± 8 correct and 200 ± 4 incorrect trials remained for the II task after artifact rejection.

Subjective Assessment of Mental Workload. The subjective experience of mental effort was quantified via the *Workload Profile (WP)* (Tsang and Velazquez, 1996), a psychometric instrument that indexes the subjective expression of mental effort along eight dimensions (perceptual/central processing, response processing, spatial processing, verbal processing, visual input modality, auditory input modality, manual output modality, speech output modality). The WP has been shown to be a highly valid, sensitive, and diagnostic index of mental workload that is well suited to assess the different cognitive demands, attentional resources, and difficulty levels of cognitive and motor tasks (Valdehita et al., 2004). Each participant's WP dimension scores were added to yield a global workload score. Given below are the instructions and ratings table utilized in the present study.

Workload Profile. Please rate the proportion of attentional resources (mental workload) you used for each task that you performed today on a scale from 0 to 1. For each task, you will provide a rating for eight different dimensions of mental workload described below:

1. Stages of processing

(1) Perceptual & Central processing. These are attentional resources required for activities like perceiving (detecting, recognizing, and identifying objects), remembering, problem-solving, and decision making.

(2) Response processing. These are attentional resources required for response selection and execution. For example, there are three foot pedals in a standard shift automobile; to stop the automobile, we have to select the appropriate pedal and step on it.

2. Processing codes

(1) Spatial processing. Some tasks are spatial in nature. Driving, for example, requires paying attention to the position of the car, the distance between the current position of the car and the next stop sign, the geographical direction that the car is heading, etc.

(2) Verbal processing. Other tasks are verbal in nature. For example, reading involves primarily processing of verbal, linguistic materials.

3. Input modality

(1) Visual processing. Some tasks are performed based on the visual information received. For example, playing basketball requires visual monitoring of the physical location & velocity of the ball. Watching TV is another example of a task that requires visual resources.

(2) Auditory processing. Other tasks are performed based on auditory information. For example, listening to the person on the other end of the telephone is a task that requires auditory attention. Listening to music is another example. Note that spatial information may be processed visually or auditorily. For example, you can get to a new restaurant by following a map (visual processing) or by following the directions spoken by your friend (auditory processing). Similarly, verbal information may be processed visually or auditorily. Listening to the news on the radio requires auditory processing of verbal materials; reading the news from the newspaper requires visual processing of verbal materials.

4. Output modalities

(1) Manual responses. Some tasks require considerable attention for producing the manual response as in typing or playing a piano.

(2) Speech responses. Other tasks require speech responses instead. For example, engaging in a conversation requires attention for producing the speech responses.

Workload Dimensions								
	Stage of Processing		Code of Processing		Input Modality		Output Modality	
Task	Perceptual & Central	Response	Spatial	Verbal	Visual	Auditory	Manual	Speech
1								
2								
3								

Note:

- A rating of 0 indicates a workload dimension required no attention for a given task
- A rating of 1 indicates that a workload dimension required maximum attention for a given task
- A rating of 0.5 indicates that a workload dimension required a degree of attention located halfway between zero & maximum attention for a given task.

Analytical Methods – Technical Details

Global Brain Free Energy Difference Quantification. The SVM algorithm was implemented using radial basis kernel functions with Baye's optimized box constraint and kernel scale parameters (uniform prior distributions ranging over [1e-5, 1e5]), and standardized predictor variables. SVM classification was implemented in MATLAB 2017b using the *fitcsvm*, *bayesopt*, *cvpartition*, and *fitSVMPosterior* functions; K-means clustering was achieved using the *kmeans* function. As SVM classification was performed using a combined stratified cross-validation and bootstrapping procedure, any distortions arising from data attrition (Pereira et al., 2009) were likely minimal because on average the number of post-artifact-rejected trials did not differ between stimulus categories (RB Task: $t(47) = 0.50$, $p > .620$; II Task: $t(47) = 0.41$, $p > .682$), nor did the ratio of incorrect-to-correct numbers of trials change from pre- to post-artifact rejection (RB Task: $t(47) = 0.24$, $p > .810$; II Task: $t(47) = 1.02$, $p > .314$).

It should also be noted that CSP feature extraction was not incorporated into the SVM cross-validation procedure and thus tested trials were transformed via a CSP matrix computed from all trials, rather than only the trials used to train the SVM classifier. Although this limitation could potentially increase the generalization error of the classifications (Blankertz et al., 2008), such an increase was likely minimal for two reasons. First, CSP patterns were computed on the basis of reported perceptions but used to predict a different set of class labels reflecting stimulus categories. Second, the tested trials for a given cross-validation fold only contributed 10% of the total trials entering into the computation of the CSP matrix and thus likely had a marginal influence on the averaged covariance matrices computed by the CSP algorithm. Moreover, any generalization error is not a major concern for the present study because the classifier's purpose was to compute conditional probabilities on the basis of diagnostic brain states occurring during a single task session and not future task sessions.

Resource Allocation Parameter Estimation. Resource parameter differences were not due to differences in the utility of perceptual differences across or within tasks. On average, the utilities used to compute the resource parameter did not differ between tasks or between perceptual decisions for either task as assessed via nonparametric, permutation-based two-way repeated-measures ANOVA: Categorization Task main effect, $F(1,47) = 0.027$, $p< 0.874$, $\eta_p^2 = 0.001$; Perceptual Decision main effect, $F(1,47) = 0.094$, $p< 0.776$, $\eta_p^2 = 0.003$; Categorization Task x Perceptual Decision interaction, $F(1,47) = 0.840$, $p< 0.370$, $\eta_p^2 = 0.018$.

Influence of Classifier Performance on Free Energy Estimation

A critical issue in establishing the validity of the free energy measure proposed in this paper is establishing the degree to which the measure is dependent on the quality or performance of the classifiers used to estimate $Q(v|\mu,m)$ from the EEG data. The first issue to note here is that a classifier is limited by the stimulus encoding quality of the brain responses to which it is applied. Clearly, a poor quality decoder applied to brain responses will classify EEG trials close to chance. However, a perfect classifier applied to brain responses will not classify trials with any greater accuracy than the brain itself. This is because the classifier is predicting trials on the basis of the brain responses, not direct information about the trials themselves. It is impossible for the decoder to reach a 100% classification of trials in this situation because the brain does not reach this level of accuracy itself! Human brains make mistakes and misclassify trials; a perfect decoder will reflect these mistakes.

Thus the real question to be answered here is, what is the effect of the shape of the $Q(v|\ \mu,m)$ distribution on the proposed free energy measure? How does the free energy measure change when the distribution is uniform (reflecting chance decoding by the brain and/or the classifier) or when the distribution exhibits non-uniformity by possessing distinct modes with small variances (reflecting

good decoding by the brain and/or the classifier)? This answer was addressed by performing a direct calculation of free energy using the optimum generative model distribution $P(o,v|M)$ and $Q(v|\mu,m)$ distributions with three different hypothetical cases of uniformity/modalness (see Tables S1 – S3, below). An Excel spreadsheet implementing the free energy calculations reported in these tables is available for inspection via the Texas Data Repository at https://dataverse.tdl.org/dataverse/info_fe_eeg. (Note that the delta function form of the optimum generative model distribution is approximated in these spreadsheets in order to avoid infinities from the free energy constituent logarithm functions.) Increasing deviations from uniformity by a distribution could reflect an increase in brain encoding of stimuli, an increase in classifier performance, or both. These calculations show that as the $Q(v|\mu,m)$ distribution deviates from nonuniformity, total free energy increases and free energy differences are accentuated between matching ($o = \mu = 1$; $o = \mu = 2$) and mismatching ($o = 1, \mu = 2$; $o = 2, \mu = 1$) optimum category perceptions and brain perceptual encoding states. These results suggest that the better quality the classification, the more sensitive the free energy measure is to the brain's encoding of stimulus category perceptions (assuming that the brain is encoding these states sufficiently in the first place). A poor quality classifier will decrease the sensitivity of this free energy measure, but if free energy differences are still observed in this case, then such findings may be considered to be conservative measurements of brain free energy.

In the present study, strong efforts were made to ensure high performance for the classifiers used to estimate $Q(v|\mu,m)$. Classification was based on maximally-informative CSP-extracted EEG features that were discriminative for each category perception o. The entire set of CSP spatial patterns was used to extract EEG features for classification, ensuring all of the relevant discriminatory information was encoded in the EEG features; typically only a few patterns are used for this purpose (Koles, 1991; Müller-Gerking et al., 1999; Ramoser et al., 2000). Moreover, the SVM algorithms were implemented using radial basis kernel functions with Baye's-optimized box constraint and kernel scale parameters, ensuring that the classifier was as discriminative as possible. Furthermore, the present observations suggest that classifier performance should not be a concern in the estimation of brain free energy from the data reported here. Classification accuracy was high for the K-means clustering classifiers used to label the category perceptions on each trial in order to sort and average the conditional probabilities computed from the SVM estimates. The accuracy of the SVM algorithms used to directly compute $Q(v|\mu,m)$ was lower, but it was also comparable to accuracy rates for participant behavior. This suggests that this classifier accurately estimated the conditional probability of a category label given brain state representations of each participant's category perceptions.

A more direct assessment of classifier performance would be to compare the free energy computed using the classifier estimate of $Q(v|\mu,m)$ to free energy computed using an estimate calculated directly from behavioral categorization performance, where in this case $Q(v|\mu,m) \equiv Q(v|d,m)$ and d indicates a behaviorally-indicated category perception decision. Per the above analysis of the effect of decoding performance on free energy estimation, if the classifier's decoding performance is worse than the brain's performance, then 1) the classifier-based estimates of ΔF_{Total} should be lower in magnitude than the behavior-based estimates of ΔF_{Total}, and 2) the classifier-based estimates of $\Delta F(o,\mu)$ should show smaller differences between matching and mismatching (o, μ) states, than the behavior-based $\Delta F(o,d)$ estimates for the corresponding (o,d) states. Table S4 shows the free energy computed from the behavior-based estimate of $Q(v|d,m)$ calculated from the relative frequencies of states v given behaviorally-indicated decisions d across trials. This estimate reproduced the basic pattern of classifier-based brain free energy estimates reported in the main text; compare Tables S4 and S5 to main text Table 3 and the ANOVA results reported in the main text section Results: Global Brain Free Energy Differences. A statistical comparison between the two types of free energy estimates (Table S5) showed that ΔF_{Total} was significantly larger for the

behavior-based estimate than classifier-based estimate. However, the differences between the two ΔF_{Total} estimations were very small (~ 1 – 2%) for both categorization tasks, suggesting that the measures yielded near-equal performance in estimating ΔF_{Total}.

The statistical comparison between the free energy difference estimates $\Delta F(o,\mu)$ and $\Delta F(o,d)$ showed that compared to the classifier-based estimate, the behavior-based estimate was larger for mismatching *(o,d)* states and smaller for matching states relative to the corresponding *(o,μ)* states of the classifier-based estimate. The differences between $\Delta F(o,\mu)$ and $\Delta F(o,d)$ for each individual state pair were non-negligible (RB Task: ~ 8 – 11% discrepancy; II Task: ~ 9 – 19% discrepancy), with larger overall $\Delta F(o,d)$ differences between matching and mismatching *(o, d)* states for the behavior-based measure than for the *(o,μ)* states of the classifier-based measure (RB Task: ~ 31% discrepancy; II Task: ~ 37% discrepancy). Direct inspection of the $Q(v|\mu,m)$ and $Q(v|d,m)$ estimates for each participant revealed the reason for the performance difference between the classifier-based ΔF_{Total} and $\Delta F(o,\mu)$ estimates. The less accurate encoding of the classifier-based estimate exacerbated $Q(v|\mu,m)$ for mismatching *(v,μ)* states while decreasing $Q(v|\mu,m)$ for matching *(v,μ)*states to an equal degree relative to the *(v,d)* states of the behavior-based $Q(v|d,m)$. This translated into an equal reduction of classifier-based $\Delta F(o,\mu)$ for mismatching *(o, μ)* states and increase of classifier-based $\Delta F(o,\mu)$ for matching states relative to the corresponding states of the behavior-based $\Delta F(o,d)$ estimate. Thus when free energy is summed across all states, the corresponding free energy changes still add up to produce a ΔF_{Total} magnitude that is nearly equal to that seen for the behavior-based measure. Nevertheless, though the classifier-based $\Delta F(o,\mu)$ measure performed worse than the behavior-based $\Delta F(o,d)$ measure, the former still demonstrated statistically-significant differences between matching and mismatching *(o, μ)* states (see main text Results: Global Brain Free Energy Differences section). This means that the classifier-based measure is a conservative estimator of $\Delta F(o,\mu)$ and provides a valid basis for drawing conclusions about the $\Delta F(o,\mu)$ differences observed in the present study.

Taken together, the results presented in this section indicate that the classifiers used in the present study were sufficiently sensitive to probe the statistics of the relevant brain states *and* that the brains of the participants were able to sufficiently distinguish among the stimulus categories (as evidenced by the participants' above-chance categorization performance). Although the behavior-based free energy measure performed better at estimating brain free energy than the classifier-based measure, the latter was used here because doing so avoids any possible statistical circularity that may arise when relating the ζ parameter estimates to brain free energy when both are estimated directly from behavioral data. Nevertheless, an important topic for future research is to determine if other classifier algorithms and/or classification procedures would yield more accurate estimates of $Q(v|\mu,m)$ and brain free energy.

Table S1. $Q(v|\mu,m)$ uniform

		$v = 1$	$v = 2$		ΔF States	ΔF	ΔF_{Total}
**P(o,v)**	$o = 1$	0.499	0.001		$F(o=1,\boldsymbol{\mu}=1)$	3.108	12.43
	$o = 2$	0.001	0.499		$F(o=1,\boldsymbol{\mu}=2)$	3.108	
**Q(v |μ)**	$\mu = 1$	0.500	0.500		$F(o=2,\boldsymbol{\mu}=1)$	3.108	
	$\mu = 2$	0.500	0.500		$F(o=2,\boldsymbol{\mu}=2)$	3.108	

Table S2. *Q(v|μ,m)* moderately modal

		v = 1	*v = 2*		**ΔF States**	**ΔF**	ΔF_{Total}
**P(o,v)**	*o = 1*	0.499	0.001		*F(o=1,μ=1)*	1.44	13.20
	o = 2	0.001	0.499		*F(o=1,μ=2)*	5.17	
**Q(v |μ)**	*μ = 1*	0.800	0.200		*F(o=2,μ=1)*	5.17	
	μ = 2	0.200	0.800		*F(o=2,μ=2)*	1.44	

Table S3. *Q(v|μ,m)* highly modal

		v = 1	*v = 2*		**ΔF States**	**ΔF**	ΔF_{Total}
**P(o,v)**	*o = 1*	0.499	0.001		*F(o=1,μ=1)*	0.694	15.17
	o = 2	0.001	0.499		*F(o=1,μ=2)*	6.894	
**Q(v |μ)**	*μ = 1*	0.999	0.001		*F(o=2,μ=1)*	6.894	
	μ = 2	0.001	0. 999		*F(o=2,μ=2)*	0. 694	

Table S4. Estimated behavior-based free energy differences.

		ΔF(o=1,d=1)	*ΔF(o=1,d=2)*	*ΔF(o=2,d=1)*	*ΔF(o=2,d=2)*	ΔF_{Total}
**II Task**		1.94<br>[1.81, 2.07]	4.41<br>[4.27, 4.56]	4.48<br>[4.30, 4.64]	1.99<br>[1.87, 2.09]	12.82<br>[12.74, 12.89]
**RB Task**		2.39<br>[2.23, 2.55]	3.93<br>[3.74, 4.11]	3.93<br>[3.74, 4.12]	2.40<br>[2.24, 2.55]	12.65<br>[12.57, 12.72]

Note. 95% CIs in parentheses. Free energy differences are in units of nats.

Table S5. Omnibus ANOVAs for Behavior-Based and Classifier- vs. Behavior-Based Free Energy Differences

		**F**	**p**	η^2
**Behavior-Based**				
Task		19.61	0.001	0.30
Free Energy State		246.37	0.001	0.84
Task x Free Energy State		24.55	0.001	0.34
**Classifier vs. Behavior-Based: II Task**				
Estimation Type		132.34	0.001	0.74
Free Energy State		272.95	0.001	0.85
Estimation Type x Free Energy State		298.32	0.001	0.86
**Classifier vs. Behavior-Based: RB Task**				
Estimation Type		39.23	0.001	0.45
Free Energy State		61.60	0.001	0.57
Estimation Type x Free Energy State		95.95	0.001	0.67

Note. For all ANOVA degrees of freedom, $df_1 = 1$ and $df_2 = 47$.

Auxiliary Behavior Analysis: Between-Category Comparisons

The statistical results reported in Table S6, below, assessed between-category differences in accuracy and reaction time for each categorization task. These results were achieved using nonparametric permutation-based one-way repeated measures ANOVA with a factor of Categorization Task (II, RB). All ANOVAs were implemented via EEGLAB.

Table S6. Between-Category ANOVAs for Task Accuracy and RTs

	F	p	η^2
**RB_ACC**	1.88	0.541	0.54
**II_ACC**	0.28	0.761	0.76
**RB_Correct RTs**	1.32	0.410	0.41
**II_Correct RTs**	5.77	0.079	0.08
**RB_Incorrect RTs**	1.44	0.482	0.48
**II_Incorrect RTs**	0.09	0.772	0.77

Note. For all ANOVA degrees of freedom, df_1 = 1 and df_2 = 47.

References

Blankertz, B., Tomioka, R., Lemm, S., Kawanabe, M., and K.-R., M. (2008). Optimizing Spatial Filters for Robust EEG Single-Trial Analysis. *IEEE Signal Processing Magazine* 25, 41–56. doi: 10.1109/MSP.2008.4408441.

Dayan, P., Hinton, G., Neal, R.M., and Zemel, R.S. (1995). The Helmholtz machine. *Neural Computation* 7**,** 889–904. doi: 10.1162/neco.1995.7.5.889.

Doya, K., Ishii, S., Pouget, A., and Rao, R.P. (2007). *Bayesian Brain: Probabilistic Approaches to Neural Coding.* Cambridge, MA: MIT Press.

Friston, K. (2012). A free energy principle for biological systems. *Entropy* 14**,** 2100–2121. doi: 10.3390/e14112100.

Gershman, S.J. (2019). What does the free energy principle tell us about the brain? *arXiv* [Online], 1901.07945v5. Available: https://arxiv.org/abs/1901.07945.

Knill, D.C., and Pouget, A. (2004). The bayesian brain: the role of uncertainty in neural coding and computation. *Trends in Neurosciences* 27**,** 712–719. doi: 10.1016/j.tins.2004.10.007.

Koles, Z.J. (1991). The quantitative extraction and topographic mapping of the abnormal components in the clinical EEG. *Electroencephalography and Clinical Neurophysiology* 79**,** 440–447. doi: 10.1016/0013-4694(91)90163-X.

Lee, T.S., and Mumford, D. (2003). Hierarchical Bayesian inference in the visual cortex. *Journal of the Optical Society of America A: Optics, Image Science, and Vision* 20**,** 1434–1448. doi: 10.1364/JOSAA.20.001434.

Müller-Gerking, J., Pfurtscheller, G., and Flyvbjerg, H. (1999). Designing optimal spatial filters for single-trial EEG classification in a movement task. *Clinical Neurophysiology* 110**,** 787–798. doi: 10.1016/S1388-2457(98)00038-8.

Ortega, P.A., and Braun, D.A. (2013). Thermodynamics as a theory of decision-making with information processing costs. *Proceedings of the Royal Society of London A: Mathematical, Physical, and Engineering Sciences* 469**,** 20120683. doi: 10.1098/rspa.2012.0683.

Pereira, F., Mitchell, T., and Botvinick, M. (2009). Machine learning classifiers and fMRI: A tutorial overview. *Neuroimage* 45**,** S199–S209. doi: 10.1016/j.neuroimage.2008.11.007.

Perrin, F., Pernier, J., Bertrand, O., Giard, M.H., and Echallier, J.F. (1987). Mapping of scalp potentials by surface spline interpolation. *Electroencephalography and Clinical Neurophysiology* 66**,** 75–81. doi: 10.1016/0013-4694(87)90141-6.

Pio-Lopez, L., Nizard, A., Friston, K., and Pezzulo, G. (2016). Active inference and robot control: a case study. *Journal of the Royal Society Interface* 13**,** 20160616. doi: 10.1098/rsif.2016.0616.

Ramoser, H., Müller-Gerking, J., and Pfurtscheller, G. (2000). Optimal spatial filtering of single trial EEG during imagined hand movement. *IEEE Transactions on Rehabilitation Engineering* 8**,** 441–446. doi: 10.1109/86.895946.

Tsang, P.S., and Velazquez, V.L. (1996). Diagnosticity and multidimensional subjective workload ratings. *Ergonomics* 39**,** 358-381. doi: 10.1080/00140139608964470.

Valdehita, S.R., Ramiro, E.D., Garcia, J.M., and Puente, J.M. (2004). Evaluation of subjective mental workload: A comparison of SWAT, NASA-TLX, and Workload Profile methods. *Applied Psychology* 53**,** 61–86. doi: 10.1111/j.1464-0597.2004.00161.x.
